# ncRNA-mediated overexpression of ubiquitin-specific proteinase 13 contributes to the progression of prostate cancer via modulating AR signaling, DNA damage repair and immune infiltration

**DOI:** 10.1186/s12885-022-10424-7

**Published:** 2022-12-23

**Authors:** Xiaolu Cui, Hongyuan Yu, Jinlong Yao, Jinling Li, Zhenhua Li, Zhenming Jiang

**Affiliations:** 1 Department of Urology, First hospital of China Medical University, Shenyang, 110001 China; 2 Department of Urology, Shengjing Hospital of China Medical University, Shenyang, 110004 China

**Keywords:** Prostate cancer, Deubiquitinase, Immunotherapy, DNA damage response, AR signaling, ceRNA

## Abstract

Metastatic castration-resistant prostate cancer (mCRPC) is a lethal form of prostate cancer, and the molecular mechanism driving mCRPC progression has not yet been fully elucidated. Immunotherapies such as chimeric antigen receptor, T-cell therapy and immune checkpoint blockade have exerted promising antitumor effects in hematological and solid tumor malignancies; however, no encouraging responses have been observed against mCRPC. The deubiquitinase USP13 functions as a tumor suppressor in many human cancers, as it sustains the protein stability of PTEN and TP53; however, its role in prostate cancer (PCa) and involvement in DNA damage and AR signaling remain unclear. In the current study, we explored the prognostic value of USP13 in PCa based on the TCGA database, and we analyzed the expression of USP13 in PCa tissues and adjacent normal tissues based on TCGA and our cohort. The results suggested that USP13 is overexpressed in PCa tumors and has the potential to be an independent biomarker for the overall survival of PCa patients. Additionally, enrichment analysis indicated that USP13 may participate in the AR pathway and PI3k/Wnt signaling, which are closely related to PCa progression. We also observed a significant correlation between the expression of USP13 and AR-related genes, DDR genes and mismatch repair genes based on the TCGA_PRAD dataset, which further supported the critical role of USP13 in AR activation and the DNA damage response of PCa. USP13 was also found to be enriched in protein neddylation, and expression of USP13 was significantly associated with infiltration of immune cells and expression of immunomodulators. Taken together, our study revealed a key role of USP13 in contributing to PCa progression by participating in multiple oncogenic signaling pathways, the DNA damage response and the immunosuppressive tumor microenvironment. Targeting USP13 may inhibit tumor growth and provide additional benefits in cooperation with DDR inhibitors and immunotherapy.

**Supplementary Information:**

The online version contains supplementary material available at 10.1186/s12885-022-10424-7.

## Introduction

Androgen receptor (AR) is the major player in initiating and promoting prostate cancer (PCa) [1, 2]. Although evolutionary strategies such as DNA damage response (DDR) inhibitors and immunotherapies have been applied in clinical practice, androgen deprivation therapy (ADT) remains the first-line therapy for PCa. However, PCa becomes resistant to ADT treatment when it enters a later stage, castration-resistant prostate cancer (CRPC) or metastatic CRPC (mCRPC), which is a more lethal form of PCa and is more difficult to confront. Hence, uncovering the mechanisms of constitutive activation of AR signaling is an inevitable topic in the research field of PCa.

Immunotherapies such as chimeric antigen receptor T-cell (CAR-T) therapy and immune checkpoint blockade (ICB) therapy have been carried out in clinical practice and have exerted promising antitumor effects in hematological and solid tumor malignancies [3–5]. However, PCa, especially mCRPC, is not one of the successful cases [6, 7]. Clinical trials indicated that patients with mCRPC benefit very limitedly from either ICB or CAR-T therapy. The limitations of immunotherapies treating PCa include the immunosuppressive tumor microenvironment (TME) [8], low tumor mutational burdens (TMB) [9, 10] and various expressions of immune checkpoint molecules [7]. Treatments received before immunotherapy attempt also affect the responses to ICB, as some studies have revealed a significant downregulation of PD-L1 in PCa tumors after abiraterone acetate therapy in combination with prednisone [11]. In addition, PCa progression was reported to affect PD-L1 expression [12]. Therefore, it is of great importance to identify specific molecular biomarkers that could predict the responses to immunotherapy and identify specific subtypes of PCa patients who may benefit from immunological treatments.

Clinical studies have suggested that most positive responses to ICIs are limited in PCa patients with mismatch repair deficiency and/or high microsatellite instability (MSI-H) tumors [13, 14] or deficiency in other DNA damage repair genes, such as CDX12 or BRCA2 [15]. It has been identified that DDR deficiency induced by either genetic alterations or pharmacological inhibitors could improve the responses of ICB in solid tumors, including prostate cancer [16, 17]. Although the mechanisms of the DDR-ICB interaction have not been fully elucidated yet, preclinical trials combining a DDR inhibitor (DDRi) with ICB showed significant additive benefits, especially in PCa patients with DDR gene deficiency [18, 19]. Mutations in DDR genes and dysregulation of the DNA damage response have been associated with DNA errors, high tumor neoantigen expression and potential ICB responses [14, 20]. All this evidence supports the critical role of DDR gene deficiency in predicting and enhancing the efficacy of immunotherapy in PCa patients.

Ubiquitin-specific proteinase 13 (USP13) belongs to the deubiquitinating enzyme (DUB) superfamily and has been well characterized to interact with and deubiquitinate tumor suppressors such as P53 [21], PTEN [22] and MITF [23], thereby modulating their protein activities. Hence, USP13 functions as a pivotal cancer silencer in most human cancers, including bladder cancer [24], melanoma [23] and breast cancer [22]. Additionally, USP13 is reported to improve the DNA damage response by recruiting the RAP80-BRCA complex to DNA damage sites in ovarian cancer, indicating that USP13 is a potential target to enhance the effectiveness of DDR inhibitors in cancers [25]. In addition, USP13 negatively modulates antiviral immunity by deubiquitinating STING, and targeting USP13 consequently triggers STING-interferon signaling and strengthens innate immunity [26]. Therefore, USP13 has the potential to regulate the response to immunotherapy and DDRi therapy. Nonetheless, to date, little is known about the biological function and immunity-related mechanisms of USP13 in prostate cancer.

In the current study, we investigated the prognostic role of USP13 in prostate cancer and explored the underlying biological functions of USP13 in driving PCa progression. Furthermore, we analyzed the association between expression of USP13 and key DDR genes, mismatch repair genes and AR-related genes. We also unearthed a potential lncRNA-mediated ceRNA regulatory network of USP13 in PCa. Our data indicated that USP13 is a potential independent biomarker for predicting the prognosis of PCa patients and that targeting USP13 might suppress the activity of AR signaling and improve the effectiveness of DDR inhibitors and ICB against CRPC.

## Materials and methods

### Clinical prostate cancer tissue samples

For the use of clinical materials for research purposes, prior patients’ written consent and approval were obtained from the First Affiliated Hospital of China Medical University. The prostate tumor specimens and clinicopathological information were obtained from 10 prostate cancer patients who underwent radical cystectomies at the Department of Urology, the First Hospital of China Medical University, from 2020 to 2021. The tissue specimens were harvested and then immediately frozen in liquid nitrogen and stored at − 80 °C. Histologically, the tumors were classified according to the 2016 World Health Organization histologic classification of tumors of the urinary system and male genital organs and were staged using the 2002 American Joint Committee on Cancer system. The use of the clinical specimens was approved by the ethics committee of the First Hospital of China Medical University (# AF-SOP-07-1.1-01).

### Quantitative real-time PCR (qRT-PCR)

The protocol of RNA extraction and qRT-PCR was performed as described before. Total RNA was extracted from clinical tissue samples (10 mg/sample) using TRIzol reagent (Invitrogen). Reverse transcription was subsequently performed using random primers from PrimeScript™ RT Master Mix (Takara Biotechnology, China). USP13 expression was measured using SYBR® Premix Ex Taq™ (Takara Biotechnology, China) and on a LightCycler™ 480 II system (Roche, Basel, Switzerland). GAPDH was used as a house keeping gene. The relative gene expression was calculated using the 2-ΔΔCt method. The experiments were repeated three times independently. Primers for mRNAs were as follows:

GAPDH-fw: 5′- TGTGGGCATCAATGGATTTGG -3′;

GAPDH-rev: 5′- ACACCATGTATTCCGGGTCAAT -3′;

USP13-fw: 5′ – TCTCCTACGACTCTCCCAATTC -3′;

USP13-rev: 5′- CAGACGCCCCTCTTACCTTCT − 3′.

### Data collection and processing

The gene expression profiles and clinical information data in each tumor and normal tissue samples were obtained from the GTEx database (https://gtexportal.org/), TCGA database (https://portal.gdc.cancer.gov/) and GEO database (https://www.ncbi.nlm.nih.gov/geo/). Seventeen types of cancers were included in the expression analysis with TCGA, and thirty-three types of cancers were included in the gene expression analysis with TCGA integrated with GTEx. Single cell RNA sequencing data from GEO database (GSE137829, GSE141445, GSE143791, GSE150692 and GSE172301) was used to analyze the expression of USP13 in single cell types.

RNA sequencing data of PCa were obtained from the TCGA database and GTEx database. A total of 496 PCa tissue samples and 152 normal prostate epithelial tissue samples were used in this study. Clinical data of the patients was also originated from TCGA database. RNA sequencing data was analyzed using R software (v4.1.3, R core team, March.10th.2022). DESeq2 package (v1.36.0, Michael Love, March.15th.2022) was used to normalized gene expression, and read counts were normalized to Transcripts Per Million (TPM). This study complied with the publication guidelines provided by TCGA.

### Kaplan–Meier analysis

The Kaplan–Meier (KM) method was used to analyze the correlation between gene expression and survival of PCa patients. The prognostic values of USP13 across cancers were assessed according to overall survival (OS) using Kaplan–Meier plotter. Kaplan–Meier analysis was conducted based on RNA sequence datasets as well as clinical survival data of PCa patients from TCGA. The cutoff values for USP13 expression were set to match the best analysis results. The KM analysis and KM curve were calculated by R software (v4.1.3, R core team, March.10th.2022), survminer package (v0.4.9, Alboukadel Kassambara, March.9th.2021) and survival package (v3.3–1, Terry M Therneau, March.3rd.2022).

### Immune infiltration and tumor immune estimation resource (TIMER)

The immune infiltration signature of USP13 was analyzed by TISIDB [27] and tumor immune estimation resource (TIMER) [28]. TISIDB is a web portal for tumor and immune system interactions that integrates multiple heterogeneous data types. Associations between the expression/methylation status of USP13 and the enrichment of twenty-eight tumor-infiltrating lymphocytes (TILs) were analyzed by TISIDB. The TIMER web server is a comprehensive resource for the systematic analysis of immune infiltrates across diverse cancer types, and the abundances of six immune infiltrates (B cells, CD4+ T cells, CD8+ T cells, neutrophils, macrophages, and dendritic cells) were estimated by the TIMER algorithm. The TIMER database was used to evaluate the correlation between USP13 expression and the infiltration level of six immune infiltrates.

### UALCAN

UALCAN (http://ualcan.path.uab.edu/) is a web-based tool that provides in-depth analyses of transcriptome data from TCGA and MET500 data [29]. In this study, UALCAN was used to investigate the association between the protein expression of USP13 and clinicopathological parameters (normal against tumor and tumor stages) and the promoter methylation level of USP13 in cancers.

### Tumor immune single-cell hub 2 (TISCH2)

scRNA-seq databases from GSE137829, GSE141445, GSE143791, GSE150692 and GSE172301 were analyzed and visualized using TISCH2 tool. TISCH2 is a web-based tool focused on analysis of tumor microenvironment basing on scRNA-seq datasets. It provides cell-type annotation and RNA expression profile at single-cell level across a variety of cancer types.

### The human protein atlas

Expression of USP13 in single cell types and cell lines was analyzed using The Human Protein Atlas tool. The Human Protein Atlas is a web-based tool to analyze human proteins in cells, tissues, and organs using integrated omics technologies. In this study, the single cell type section was used to analyze the expression of USP13 in single human cell types based on scRNA-seq.

### The encyclopedia of RNA Interactomes (ENCORI)

The Encyclopedia of RNA Interactomes (ENCORI) is an open-source platform for studying miRNA-ncRNA, miRNA–mRNA, ncRNA-RNA, RNA–RNA, RBP-ncRNA and RBP-mRNA interactions from CLIP-seq, degradome-seq and RNA–RNA interactome data [30]. In this study, the interactions of USP13-miRNAs and miRNA-lncRNAs were analyzed by ENCORI. The miRNA-mRNA module was used to predict USP13-targetting miRNAs. The parameters were set as following: CLIP-data > = 3, programNum > = 1, pan-cancer > = 5, degradome-data > = 3, miRNA: all. The miRNA-lncRNA module was used to predict miRNAs-targetting lncRNAs. The parameters were set as following: CLIP-data > = 3, pan-cancer > = 5, degradome-data > = 3.

### Gene ontology (GO) term and gene set enrichment analysis (GSEA)

To investigate the potential biological processes and pathways involved in USP13, the differentially expressed genes (DEGs) according to USP13 in prostate cancer were analyzed, and gene ontology (GO) enrichment analysis and gene set enrichment analysis (GSEA) were performed using LinkedOmics [31]. The analysis of DEGs, GO and GSEA were also visualized by LinkedOmics.

### Gene-to-gene and protein-to-protein interaction analysis

The GeneMANIA (http://www.genemania.org) database was applied to construct the gene-to-gene interaction network of USP13. The STRING database (https://string-db.org/) was applied to construct the protein-to-protein (PPIs) interaction network of USP13. The parameters for analysis using GeneMANIA and STRING database were set as default.

The top 100 similar genes of USP13 in prostate tumors were obtained from GEPIA (http://gepia.cancer-pku.cn/) [32]. Metascape [33] online database was applied to perform the pathway and process enrichment analysis and protein-protein interaction enrichment analysis of USP13. The list of 101 genes (USP13 and its 100 similar genes in PCa) was input into Metascape and analysis was performed subsequently. The network of enrichment terms and the PPI network was analyzed and visualized by Metascape.

Protein to protein interaction enrichment analysis was performed using the following databases: STRING (physical score > 0.132), BioGrid, OmniPath and InWeb_IM. The Molecular Complex Detection (MCODE) algorithm was used to identify key network components. Pathway and process enrichment analysis was then performed on each MCODE component, independently.

### cBioPortal

The cBioPortal dataset (https://www.cbioportal.org/) contains multiple data types, such as somatic mutations, DNA methylation, protein enrichment, and miRNA expression, to facilitate the study of multidimensional cancer gene datasets. The mutation of USP13 in cancers as well as the structure of USP13 were provided and visualized by cBioPortal.

## Results

### USP13 is dysregulated in human cancers

**Fig. 1:**
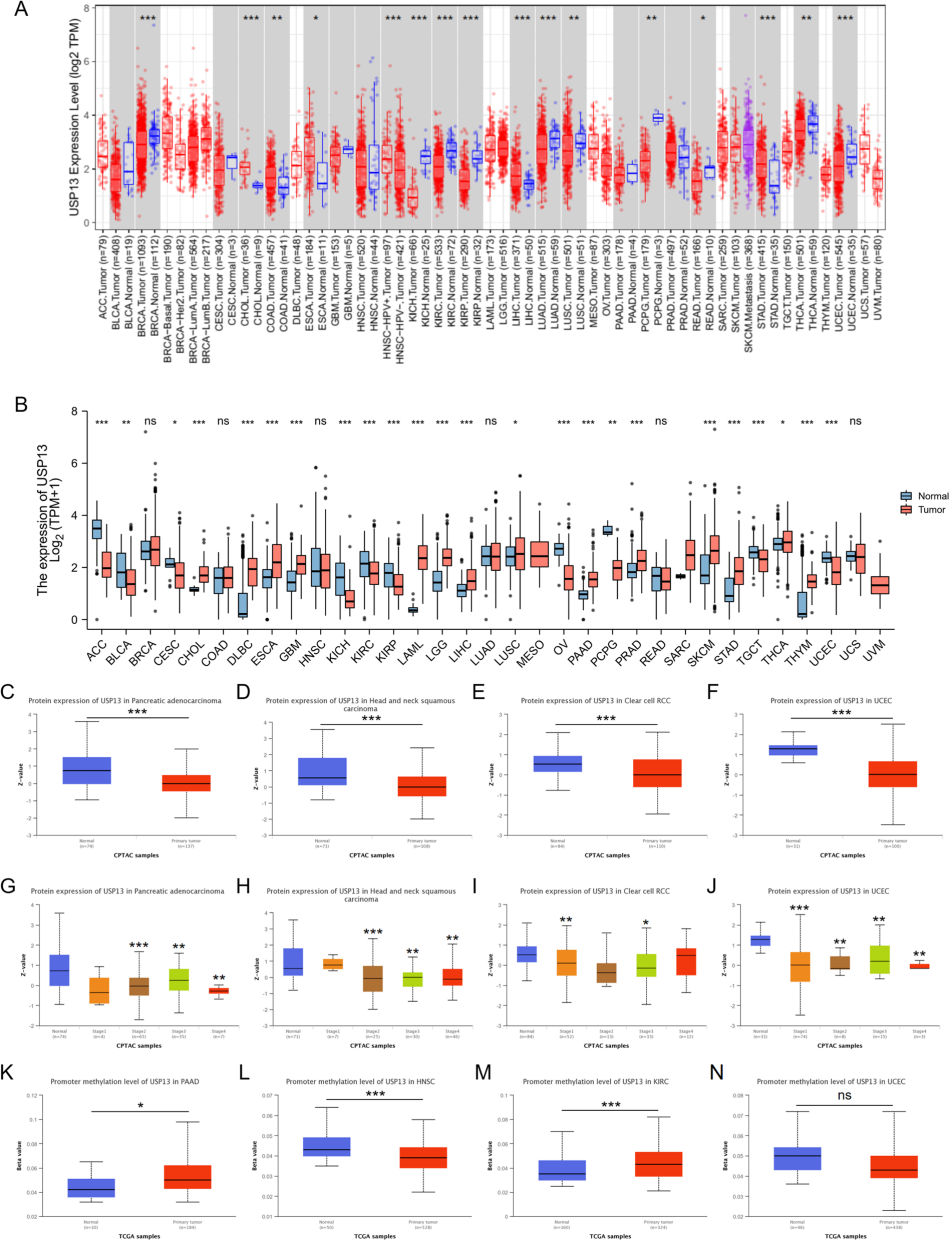
USP13 is dysregulated in human cancers

By stabilizing the expression of PTEN and P53, USP13 has been recognized as a tumor suppressor in most human cancers. Hence, we performed a pan-cancer analysis to evaluate the expression of USP13 in different cancer types based on the TCGA database. Using the TIMER tool, USP13 expression was analyzed and compared in 17 cancer types between tumor tissues and relative normal tissue samples. The results suggested that USP13 was differentially expressed between tumor and normal tissues in 15 cancer types; among them, USP13 was found to be significantly upregulated in tumors in 10 types of cancers (Fig. 1A). We further explored USP13 expression in 33 types of human cancers based on the RNA sequencing data of normal tissues from the GTEx database along with the data of normal and tumor tissues from the TCGA database. The results showed that USP13 expression was significantly elevated in tumor tissues in 10 cancer types compared with normal tissue samples (Fig. 1B). Next, we analyzed the proteomic expression of USP13 in cancers using data from the Clinical Proteomic Tumor Analysis Consortium (CPTAC) by UALCAN. The results suggested that the protein expression of USP13 is significantly elevated in tumor tissues compared with normal samples in pancreatic adenocarcinoma (PAAD), head and neck squamous carcinoma (HNSC), clear cell renal cell carcinoma (ccRCC) and uterine corpus endometrial carcinoma (UCEC) (Fig. 1C – F). Moreover, USP13 protein expression was found to be positively correlated with the progression of PAAD, HNSC, ccRCC and UCEC (Fig. 1G – J). We also analyzed the methylation of the USP13 promoter in PAAD, HNSC, ccRCC and UCEC, and the results suggested that the USP13 promoter was hypermethylated in PAAD and ccRCC tumor tissues and hypomethylated in HNSC tumor tissues compared with normal tissue samples (Fig. 1K-N).

**Fig. 2:**
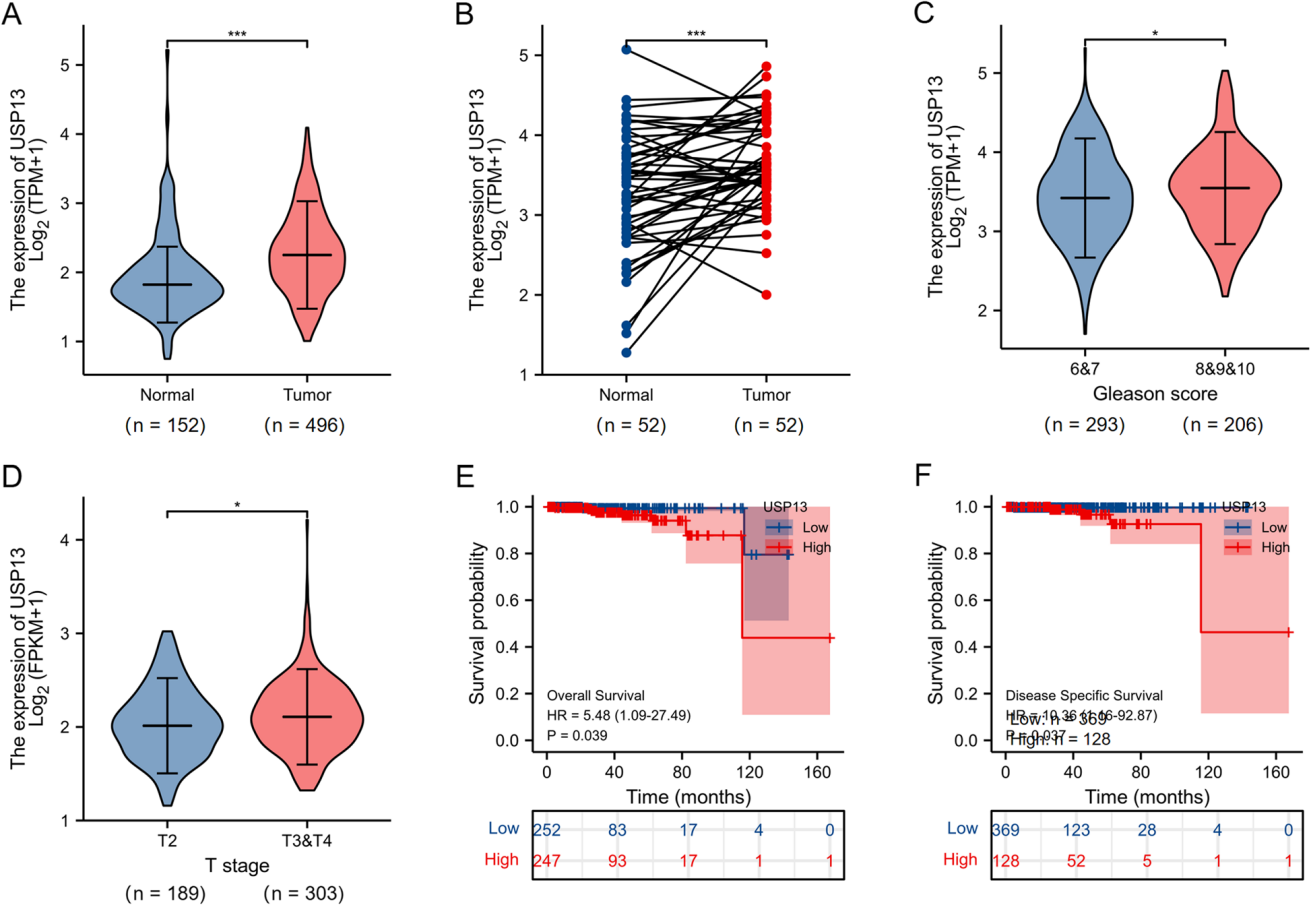
USP13 is highly expressed in PCa tumor tissues and is related to poor prognosis and progression of PCa

The prognostic role of USP13 across cancers was analyzed using Kaplan–Meier curves based on the TCGA database. USP13 expression was found to be positively correlated with the optimistic prognosis of 6 cancer types and poor prognosis of the other 10 cancer types (S Fig. 1). Data from cBioPortal showed that amplification of the USP13 gene is common in cancers, and the protein structure along with the phosphorylation, ubiquitination and methylation sites were also analyzed (S Fig. 2A and B). Collectively, dysregulation of USP13 in cancers is commonly observed, and the role of USP13 in tumorigenesis can be controversial according to different cancer types.

A. Expression of USP13 was detected in normal and tumor tissues in 15 tumor types by Gepia. B. Expression of USP13 was analyzed between normal and tumor tissues in 33 types of human cancers based on TCGA integrated with GTEx. Protein expression of USP13 was found to be downregulated in tumor tissues compared with normal tissue samples in pancreatic adenocarcinoma (PAAD) (C), head and neck squamous carcinoma (HNSC) (D), clear cell renal carcinoma (ccRCC) (E) and uterine corpus endometrial carcinoma (UCEC) (F) by UALCAN. Protein expression of USP13 was found to be positively correlated with tumor progression of PAAD (G), HNSC (H), ccRCC (I) and UCEC (J). The methylation status of the USP13 promoter was analyzed in PAAD (K), HNSC (L), ccRCC (M) and UCEC (N). ns indicates not significant, * indicates *p* < 0.05, ** indicates *p* < 0.01, *** indicates *p* < 0.001.

### USP13 is overexpressed in prostate cancer tissues and serves as an independent predictor for PCa patients

Next, we analyzed USP13 expression and its prognostic value in prostate cancer based on the TCGA database and GTEx database. The expression of USP13 was found to be significantly upregulated in 496 PCa tumor tissues compared with 152 normal tissue samples (Fig. 2A), and a similar result was observed when comparing USP13 expression in 52 pairs of prostate tumor and adjacent normal prostate epithelial tissues (Fig. 2B). The clinicopathological characteristics and USP13 expression of 499 patients with prostate cancer were obtained from TCGA, and the association of USP13 expression and clinicopathological characteristics was analyzed using the chi-square test and Fisher’s exact test (Table 1). USP13 expression was found to be associated with lymph node metastasis and Gleason’s score. We further analyzed the significance of USP13 expression in prostate progression, and the results confirmed that high expression of USP13 was correlated with a high Gleason’s score (8 &9 &10 vs 6 & 7) and advanced tumor stage (T3 & T4 vs T2) of PCa (Fig. 2C and D). Kaplan–Meier (KM) analysis showed that high expression of USP13 was positively related to poor overall survival and disease-specific survival of PCa patients (Fig. 2E and F). Cox regression univariate/multivariate analysis suggested that USP13 could be an independent predictor for the overall survival of PCa patients (Table 2). Logistic regression analysis showed that USP13 expression was correlated with lymph node metastasis (Table 3).

**Table 1:** The clinicopathological characteristics and USP13 expression of 499 patients with prostate cancer

Characteristic	Low expression of USP13	High expression of USP13	*p*
n	249	250	
T stage, n (%)			0.107
T2	103 (20.9%)	86 (17.5%)	
T3	139 (28.3%)	153 (31.1%)	
T4	3 (0.6%)	8 (1.6%)	
N stage, n (%)			**0.032**
N0	172 (40.4%)	175 (41.1%)	
N1	28 (6.6%)	51 (12%)	
M stage, n (%)			0.123
M0	225 (49.1%)	230 (50.2%)	
M1	3 (0.7%)	0 (0%)	
PSA(ng/ml), n (%)			0.243
< 4	210 (47.5%)	205 (46.4%)	
> =4	10 (2.3%)	17 (3.8%)	
Gleason score, n (%)			**0.009**
6	32 (6.4%)	14 (2.8%)	
7	125 (25.1%)	122 (24.4%)	
8	35 (7%)	29 (5.8%)	
9	56 (11.2%)	82 (16.4%)	
10	1 (0.2%)	3 (0.6%)	

Analysis of USP13 expression between M stage was performed using Fisher’s exact test, the other analysis was carried out using Chi-square’s test

**Table 2:** Univariate and multivariate Cox regression analyses of clinicopathological characteristics along with USP13 expression in PC patients

Characteristics	Total(N)	Univariate analysis		Multivariate analysis	
		Hazard ratio (95% CI)	*P* value	Hazard ratio (95% CI)	*P* value
T stage	492				
T2	189	Reference			
T3&T4	303	3.294 (0.612–17.727)	0.165		
N stage	426				
N0	347	Reference			
N1	79	3.516 (0.778–15.896)	0.102		
M stage	458				
M0	455	Reference			
M1	3	59.383 (6.520–540.817)	**< 0.001**	28.959 (2.836–295.678)	**0.005**
PSA (ng/ml)	442				
< 4	415	Reference			
> = 4	27	10.479 (2.471–44.437)	**0.001**	5.236 (1.125–24.363)	**0.035**
Gleason score	499				
6&7	293	Reference			
8&9&10	206	6.664 (1.373–32.340)	**0.019**	4.327 (0.793–23.613)	0.091
USP13	499				
Low	249	Reference			
High	250	5.439 (1.082–27.333)	**0.040**	5.439 (1.082–27.333)	**0.040**

**Table 3:** Logistics regression analyses of clinicopathological characteristics along with USP13 expression in PC patients

Characteristics	Total(N)	Odds Ratio (OR)	*P* value
T stage (T3&T4 vs. T2)	492	1.312 (0.912–1.890)	0.144
N stage (N1 vs. N0)	426	1.893 (1.145–3.189)	**0.014**
M stage (M1 vs. M0)	458	2.009 (0.191–43.406)	0.570

**Fig. 3:**
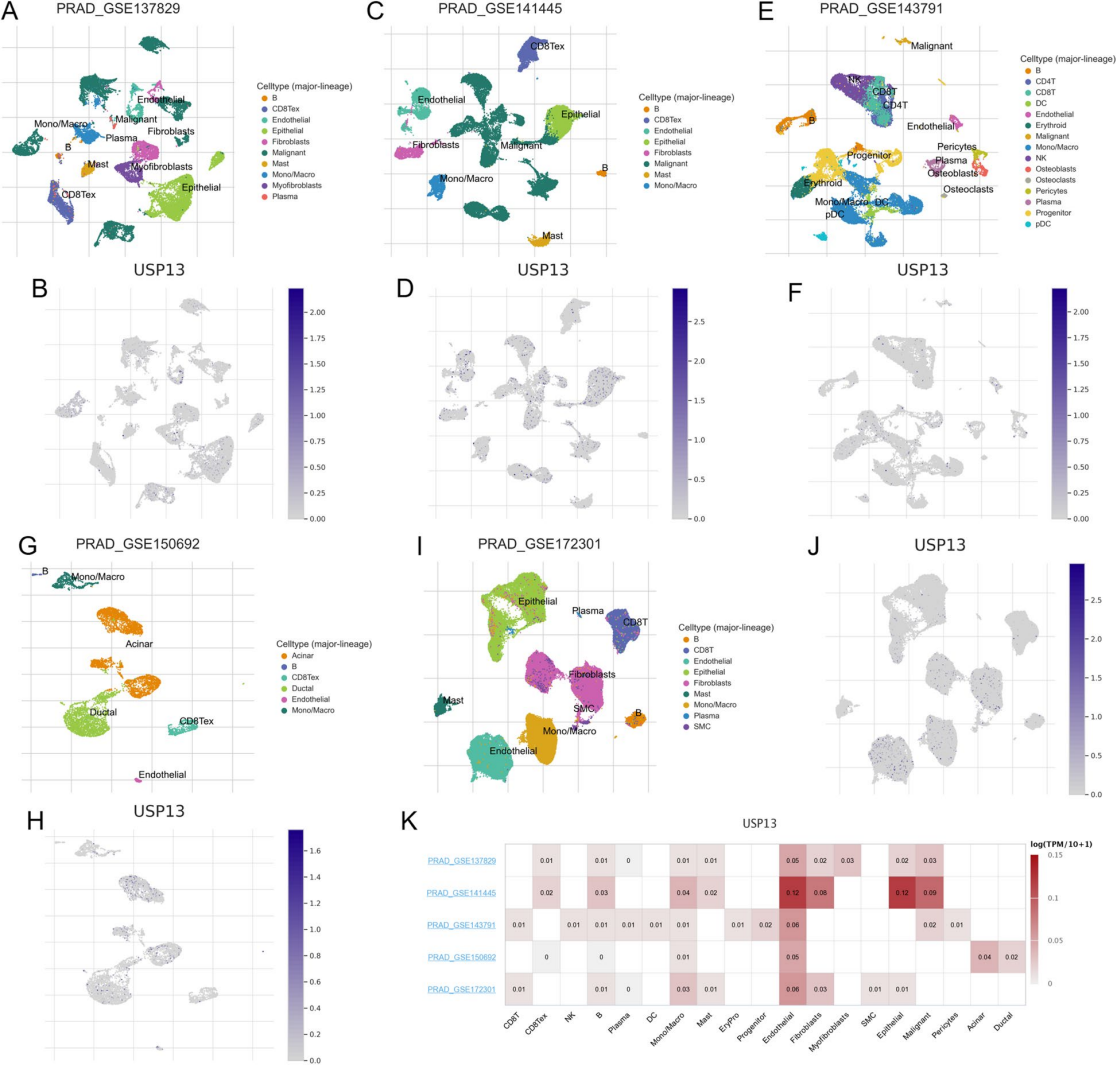
Expression of USP13 in single cell types from prostate cancer tumors

Expression of USP13 in single cell types separated from prostate cancer tumors was measured by analyzing the single cell RNA sequencing (scRNA-seq) data in GEO database. The clusters of cell types in PCa tumor microenvironment and expression of USP13 in different cell types were analyzed in scRNA-seq profiles from GSE137829 (Fig. 3A and B), GSE141445 (Fig. 3C and D), GSE143791 (Fig. 3E and F), GSE150692 (Fig. 3G and H) and GSE172301 (Fig. 3I and J). USP13 was found to be highly expressed in epithelial cells, endothelial cells, malignant cells and fibroblast cells (Fig. 3K). In addition, low expression of USP13 was observed in almost all types of immune cells. Expression of USP13 in single cell types and cell lines was analyzed by The Human Protein Atlas and demonstrated in S Fig. 3.

**Fig. 4:**
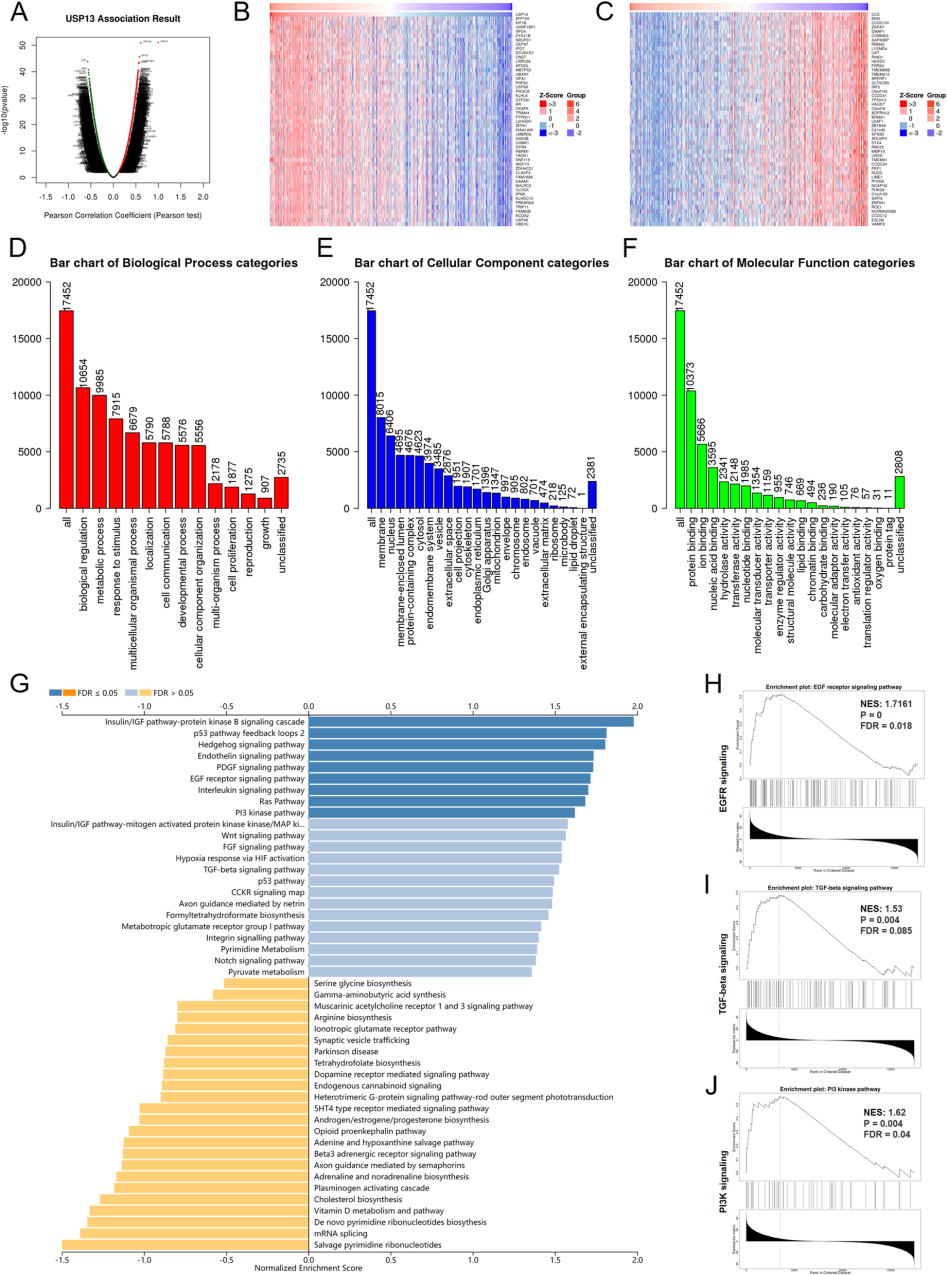
Underlying mechanisms of USP13 in prostate cancer

**Fig. 5:**
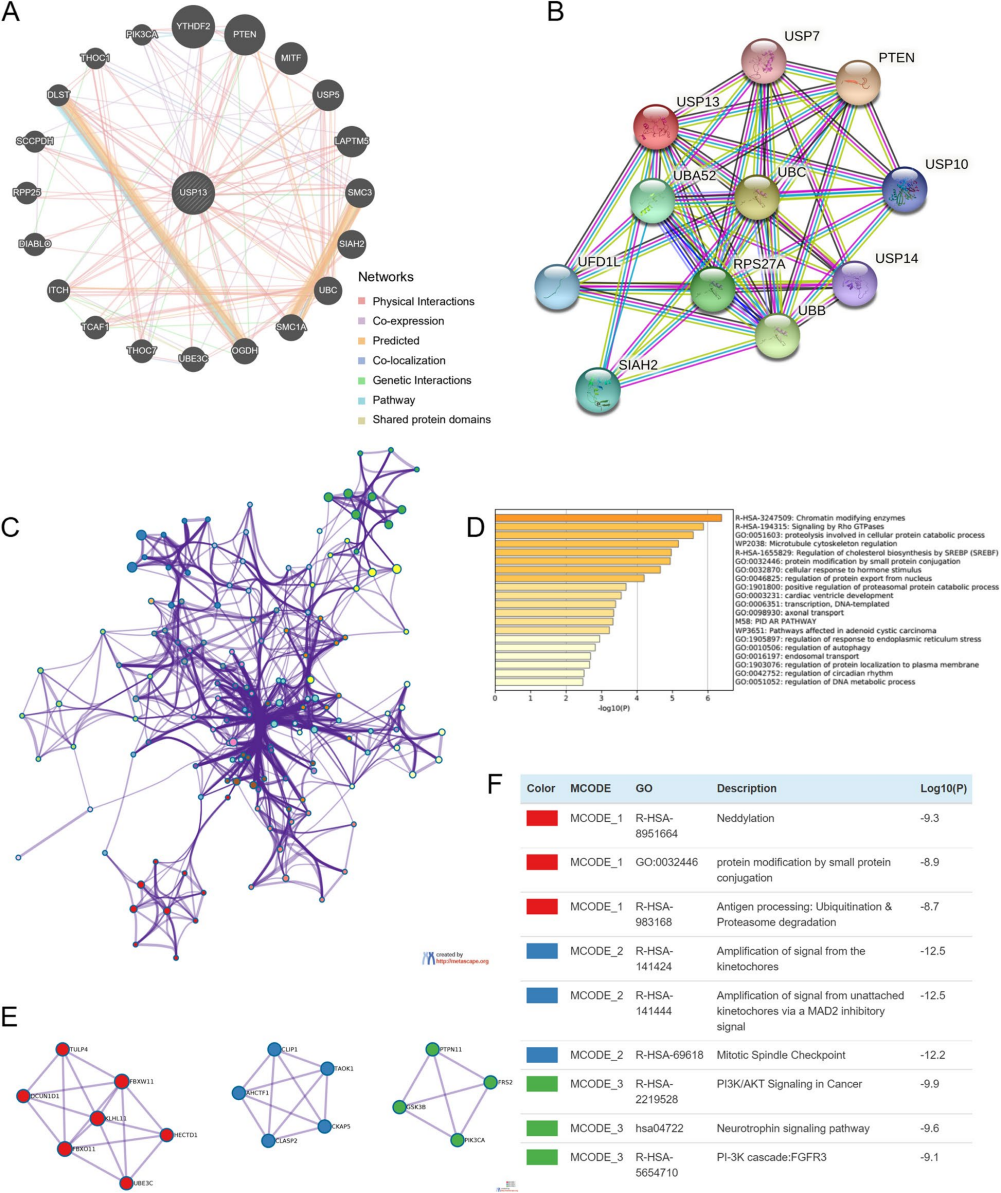
Networks of gene-to-gene and protein-to-protein interactions of USP13 in PCa

qRT-PCR was performed to evaluate expression of USP13 gene in 10 PCa tumor tissue samples. The USP13 gene expression and clinicopathological characteristics of the patients were shown in S Fig. 4A and B. The methylation level of the USP13 gene promoter was found to be significantly higher in PCa tumors, tumors without TP53 mutation and tumors with or without lymph node metastasis than in normal tissue samples (S Fig. 5A – 5C); moreover, the expression of USP13 was positively associated with methyltransferases in PCa (S Fig. 5D). Combined, the expression of USP13 was elevated in PCa tumors, which may be due to the hyper-methylated level of USP13 gene promoter, and high expression of USP13 indicated poor survival of PCa patients.

A. USP13 was highly expressed in 496 PCa tumor tissues compared to 152 normal tissues based on TCGA and GTEx databases. B. USP13 was highly expressed in 52 pairs of PCa tumor and adjacent normal tissue samples. USP13 expression was correlated with tumors of high Gleason’s score (C) and advanced stage (D). Overexpression of USP13 was significantly associated with poor overall (E) and disease-specific survival (F) of PCa patients. * indicates *p* < 0.05, ** indicates *p* < 0.01, *** indicates *p* < 0.001.

The clusters of different cell types and expression of USP13 in single cell types were analyzed basing on scRNA-seq data from GSE137829 (A and B), GSE141445 (C and D), GSE143791 (E and F), GSE150692 (G and H) and GSE172301 (I and J). K. Expression profile of USP13 in different cell types basing on scRNA-seq data from five GSE datasets was shown.

### Underlying biological functions and PPI network of USP13 in PCa

To investigate the mechanisms underlying the biological functions of USP13 in prostate cancer, differentially expressed genes (DEGs) were analyzed between PCa tumor tissues with high or low expression level of USP13 (separated by the median expression level of USP13) from the TCGA database (Fig. 4A). Analysis was conducted using LinkedOmics. The top 50 genes positively or negatively correlated to USP13 are shown in Fig. 4B and C. Gene ontology (GO) enrichment pathway analysis and gene set enrichment analysis (GSEA) were performed to evaluate the enriched pathways of DEGs. GO analysis suggested that USP13 was enriched in biological process (BP) such as biological regulation, response to stimulus, cell proliferation and growth (Fig. 4D); in terms of cellular components (CC) such as membrane, nucleus and ribosome (Fig. 4E); and in terms of molecular function (MF) categories such as protein binding, ion binding and nucleic acid binding (Fig. 4F). GSEA analysis indicated that USP13 was enriched in critical caner-driving pathways (Fig. 4G), including the EGFR pathway (Fig. 4H), TGF-beta pathway (Fig. 4I) and PI3K pathway (Fig. 4J). In addition, USP13 was found to be enriched in the hypoxia response via HIF activation, suggesting that targeting USP13 may suppress hypoxia and thereby promote immune infiltration and the response to immunotherapy in prostate cancer [34].

The networks of gene-to-gene interaction and protein-to-protein interaction were carried out using GeneMANIA and STRING database. USP13 gene was found to be physically interacted with genes such as PTEN, YTHDF2, MITF, UBC and SMC1A, and co-expressed with PIK3CA (Fig. 5A). USP13 protein was found to interact with PTEN, UBC, USP5 and SIAH2 (Fig. 5B). The top 100 most similar genes of USP13 in PCa were analyzed by GEPIA and then subjected to pathway and process enrichment analysis and protein-protein interaction enrichment analysis using Metascape. The network of enriched terms of the 101 genes is shown in Fig. 5C, and the top 20 enriched biological functions are shown in Fig. 5D. The results suggested that enriched terms were cellular response to hormone stimulus and AR pathway, indicating a potential regulatory role of USP13 in PCa. In addition, the hub genes in the PPI network were analyzed using the MCODE module, and three gene clusters were identified and shown in Fig. 5E and F, the hub genes were enriched in neddylation (red), amplification of the signal from kinetochores (blue) and PI3K/AKT signaling in cancer (green). Neddylation is a posttranslational modification in which the ubiquitin-like protein NEDD8 is added to substrate proteins and has been recognized to modulate tumorigenesis and influence components of the tumor microenvironment, such as immune cells and cancer-associated fibroblasts (CAFs). Moreover, PI3K/AKT has been documented as a hallmark that drives PCa progression. Taken together, the analysis of pathway and process enrichment and PPI enrichment further confirmed the carcinogenesis and immune-related signatures of USP13 in prostate cancer.

A. Differentially expressed genes according to USP13 in PCa were analyzed using LinkedOmics. (B) Heatmaps showing the top 50 genes positively correlated with USP13 in prostate cancer. (C) Heatmaps showing the top 50 genes negatively correlated with USP13 in prostate cancer. (D - F) Bar charts indicating enriched terms of biological process (BP), cellular component (CC) and molecular function (MF) of USP13 in PCa by GO analysis. G. Enriched biological processes and pathways of USP13 in PCa by GSEA. Three representative pathways of USP13 in PCa, namely, the EGF receptor signaling pathway (H), TGF-beta signaling pathway (I) and PI3K kinase pathway (J), are shown here. Nes indicates normalized enrichment score.

Network of gene-to-gene interactions was conducted by GeneMANIA (A), and STRING database was applied to analyze the network of PPI of USP13 (B). The top 100 similar genes of USP13 in PCa were analyzed by GEPIA, and analysis of pathway and biological process enrichment was performed by Metascape (C and D). PPI network and hub genes along with enriched terms were analyzed and visualized by Metascape (E and F).

### Associations between USP13 expression and AR signaling genes, DDR genes and MMR genes in PCa

**Fig. 6:**
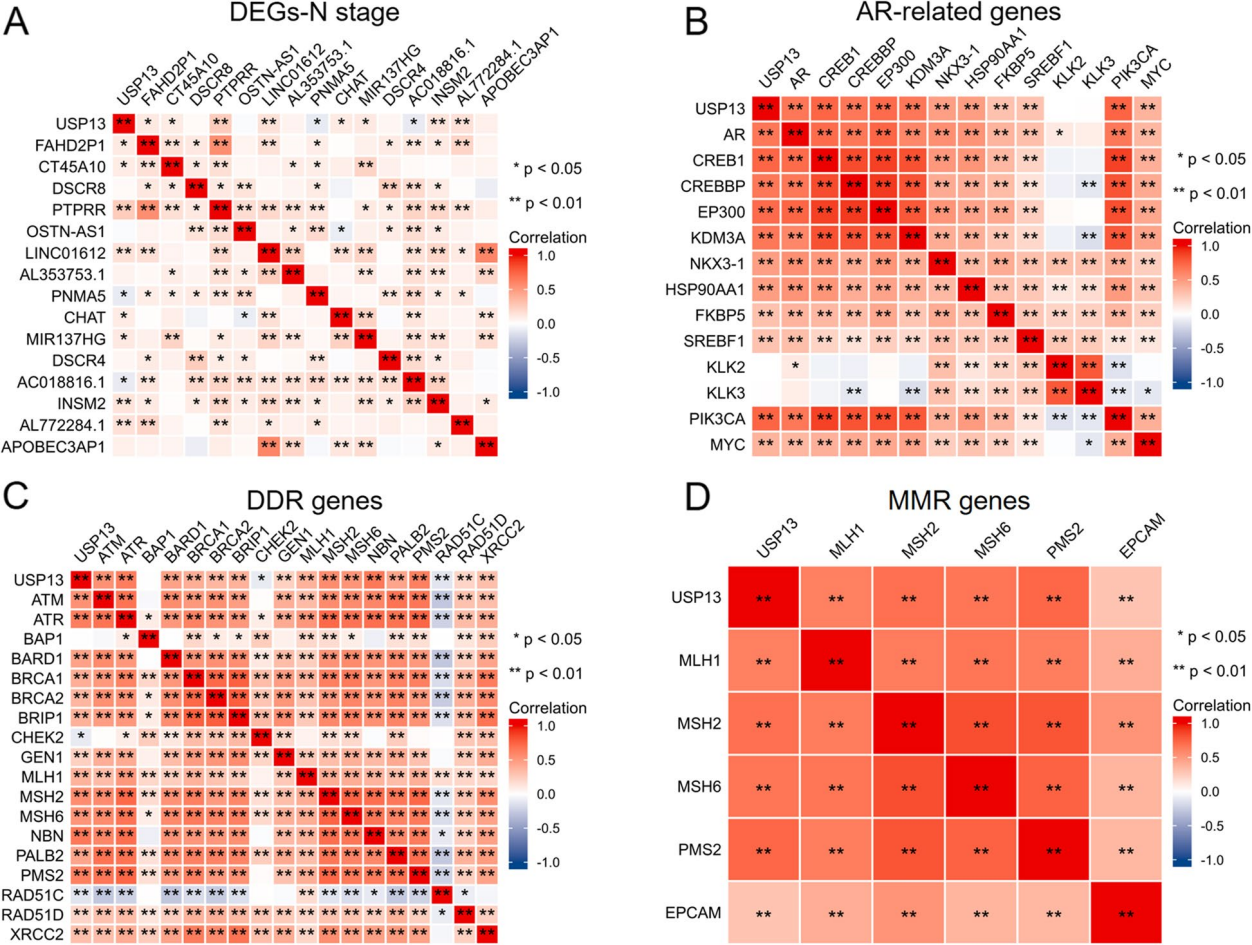
Associations between the expression of USP13 and PCa-related genes, DDR genes, MMRs and methylases in PCa based on TCGA

To further explore the potential role of USP13 in regulating the immune response, DNA damage response and AR signaling of prostate cancer cells, we analyzed the relationship between USP13 expression and key regulators of the above biological process. First, we analyzed the DEGs between normal and prostate tumor tissues (DEGs-normal vs tumor) based on TCGA-PRAD dataset (S Fig. 6A). In addition, the DEGs between prostate tumors with N0 and N1 stage (DEGs-N stage), DEGs between prostate tumors with low Gleason’s score (≤ 7) and high Gleason’s score (DEGs-Gleason’s score) (S Fig. 6B), DEGs between prostate tumors with T2 stage and T4 stage (DEGs-T stage) (S Fig. 6C) and DEGs between prostate tumors with or without metastasis (DEGs-M stage) (S Fig. 6D) were analyzed as well. The association between expression of USP13 and the top 15 DEGs of each group was evaluated by Spearman’s correlation analysis. The results indicated that, to some extent, USP13 expression was positively correlated with most DEGs from the DEG-N stage (Fig. 6A).

Next, we analyzed the association between USP13 expression and AR-related genes. AR activators and coactivators, namely, KDM3A, CREB1, CREBBP, EP300 and HSP90AA1, were selected, and AR target genes, including NKX3.1, FKBP5, SREBF1, KLK2, KLK3 and MYC, were selected. Surprisingly, USP13 expression was found to be positively correlated with most AR-related genes except for KLK2 and KLK3 (Fig. 6B), indicating that overexpression of USP13 was very likely to be related to AR activation. We then analyzed the relationship between USP13 and DNA damage repair in PCa cells. The results suggested that USP13 expression was significantly related to the expression of frequently altered DDR genes [35] (Fig. 6C) and mismatch repair (MMR) genes (Fig. 6D). Collectively, USP13 expression was significantly associated with the expression of AR activators/coactivators, AR target genes, DDR genes and MMR genes, and USP13 was expected to be correlated with the clinical progression of PCa, such as lymph node metastasis.

A. Association between the expression of USP13 and the top 15 differentially expressed genes between PCa tumors with or without lymph node metastasis. B. Association between the expression of USP13 and AR-related genes. C. Association between the expression of USP13 and DDR genes. D. Association between the expression of USP13 and MMR genes. * indicates *p* < 0.05, ** indicates *p* < 0.01.

### Association between USP13 expression and immune infiltration in PCa

**Fig. 7:**
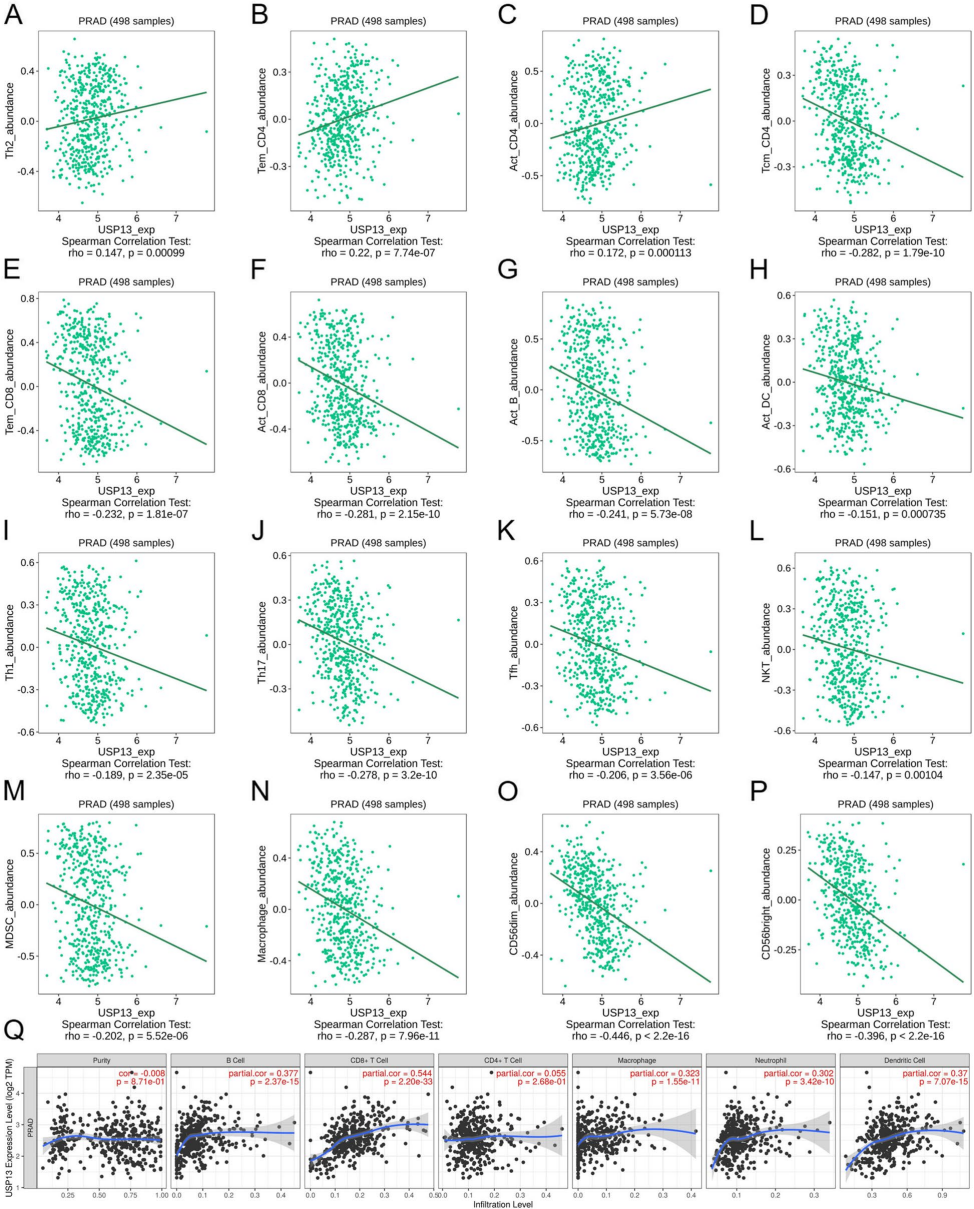
Association between USP13 expression and immune infiltration

**Fig. 8:**
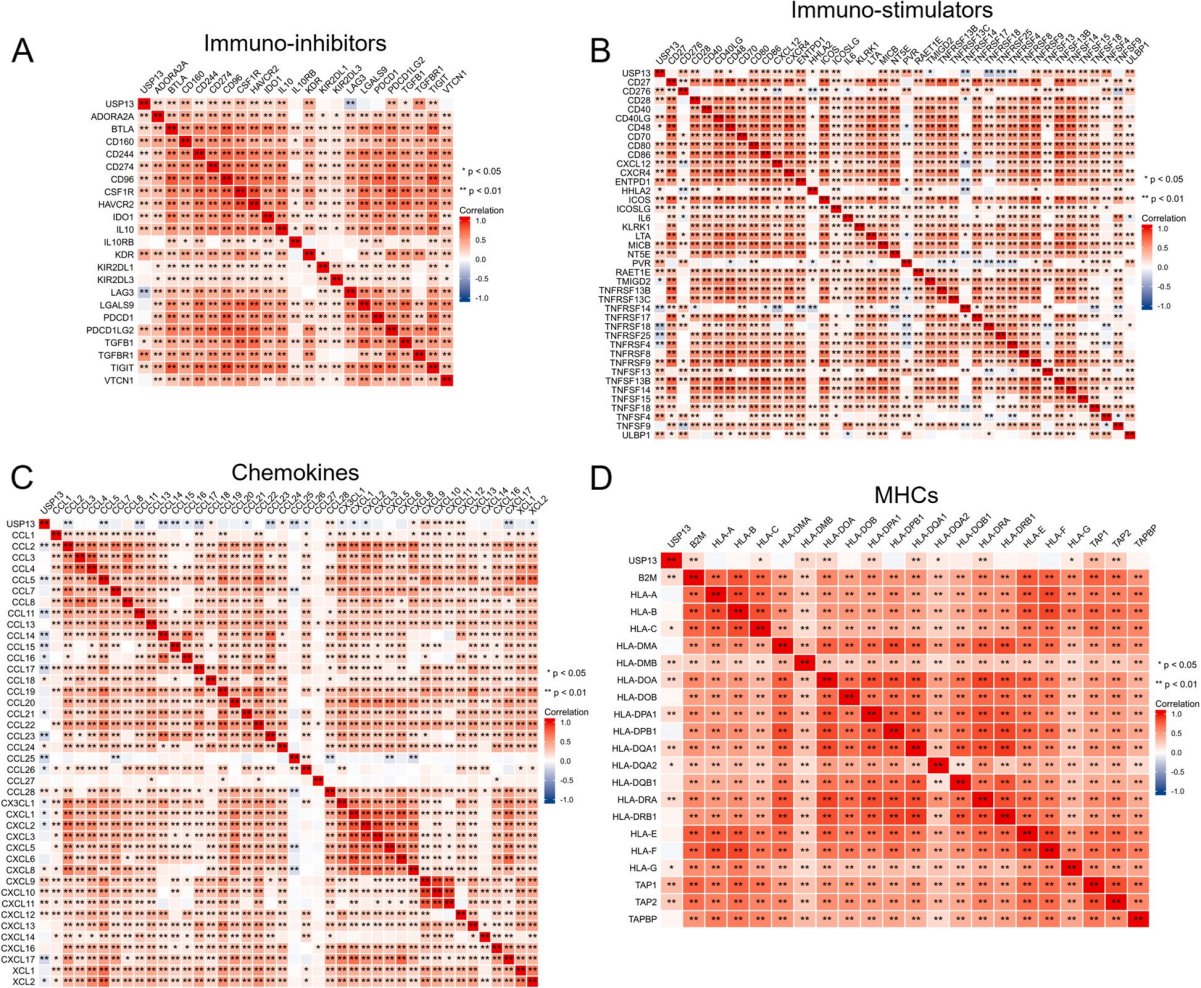
Association between USP13 expression and immunomodulators in PCa based on TCGA

Having noticed that USP13 might participate in protein neddylation and DDR of PCa cells, we then sought to explore whether USP13 affected immune cell infiltration. Correlations between USP13 expression and the abundance of immune cells were analyzed using TISIDB and TIMER. The results from TISIDB suggested that USP13 expression was positively correlated with the abundance of central memory CD4+ T cells (Tcm_CD4), type 2 T helper cells (Th2) and activated CD4+ T cells (Act_CD4) (Fig. 7A – C) but negatively correlated with the abundance of various immune cells, such as activated CD8+ T cells (Act_CD8), macrophages, activated B cells (Act_B), activated dendritic cells (Act_DC) and natural killing T cells (NKT) (Fig. 7D – P). However, the results from TIMER showed that USP13 expression was positively correlated with the infiltration levels of B cells (R = 0.377, *p* < 0.001), CD8+ T cells (R = 0.544, p < 0.001), macrophages (R = 0.323, p < 0.001), neutrophils (R = 0.302, p < 0.001) and dendritic cells (R = 0.37, p < 0.001), as shown in Fig. 7Q. We then analyzed the association between the expression of USP13 and immune inhibitors (Fig. 8A), immunostulators (Fig. 8B), chemokines (Fig. 8C) and major histocompatibility complex (MHC) (Fig. 8D) in PCa based on TCGA_PRAD. The results indicated that the expression of USP13 was very likely to positively correlate with the expression of most immune inhibitors and immune-stimulators and negatively correlate with chemokine expression. Associations between the methylation level of USP13 and immune cell infiltration are shown in S Fig. 7. Overall, USP13 was found to be a potential modulator in immune cell infiltration and the tumor microenvironment modulation in PCa.

Associations between USP13 expression and abundance of type 2 T helper cells (Th2) (A), effector memory CD4 T cells (Tem_CD4) (B), activated CD4+ T cells (Act_CD4) (C), central memory CD4 T cells (Tcm_CD4) (D), effector CD8 T cells (Tem_CD8) (E), activated CD8+ T cells (Act_CD8) (F), activated B cells (Act_B) (G), activated dendritic cells (Act_DC) (H), Type 1 T helper cells (I), Type 17 T helper cells (Th17) (J), T follicular helper cells (Tfh) (K), natural killing T cells (NKT) (L), myeloid-derived suppressor cells (MDSCs) (M), macrophages (N), CD56dim natural killer cells (CD56dim) (O), and CD56dim natural killer cells (CD56bright) (P). (Q) Association between USP13 expression and the infiltration levels of B cells, CD8+ T cells, CD4+ T cells, macrophages, neutrophils and dendritic cells were analyzed by TIMER.

A. Association between the expression of USP13 and immunoinhibitors. B. Association between the expression of USP13 and immune stimulators. C. Association between the expression of USP13 and chemokines. D. Association between the expression of USP13 and major histocompatibility complex (MHC). * indicates *p* < 0.05, ** indicates *p* < 0.01.

### Analysis of the ncRNA-mediated network of USP13 in PCa

**Fig. 9:**
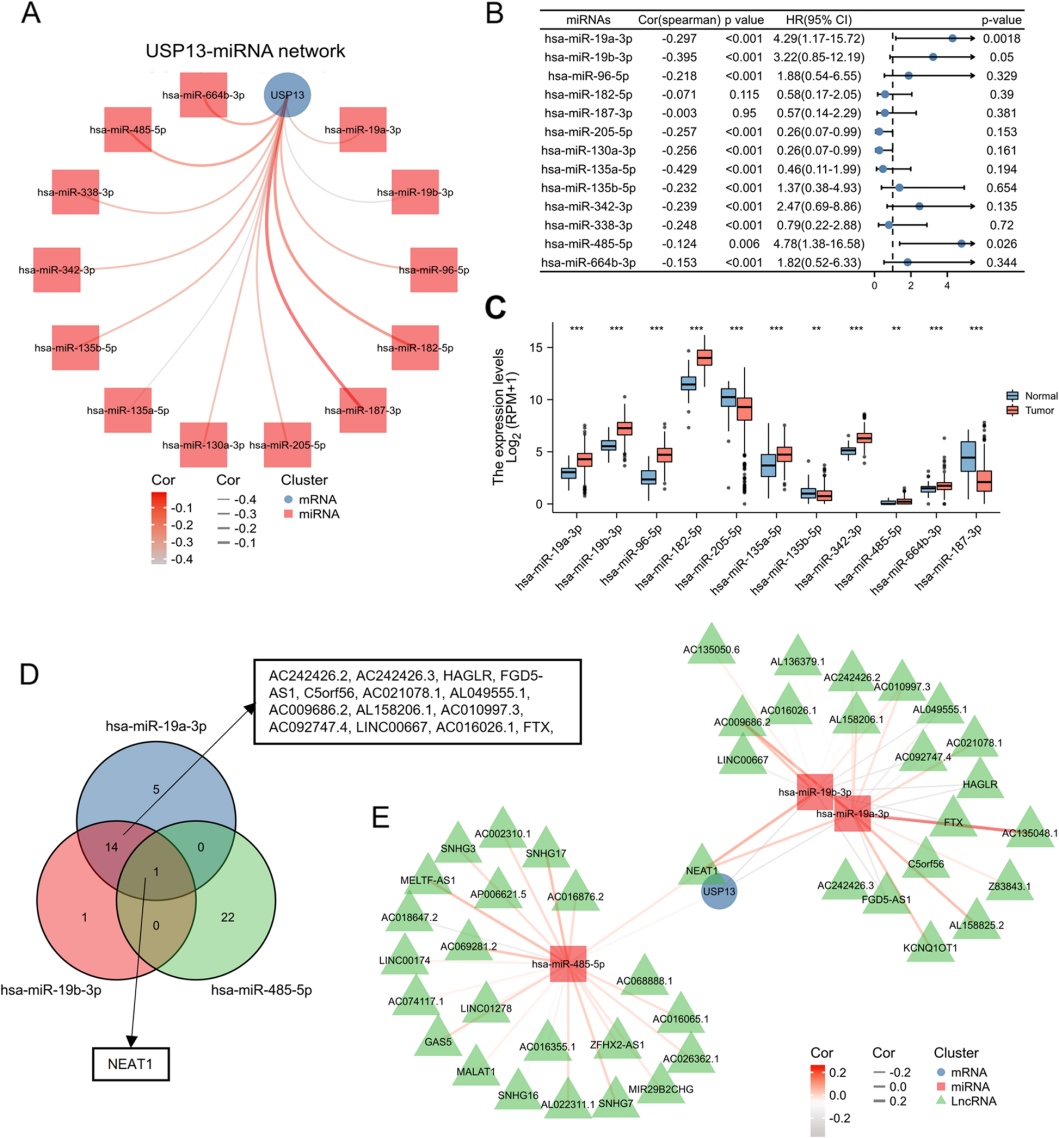
ncRNA-regulatory network of USP13 in PCa

Finally, we sought to explore the ncRNA-mediated network of USP13 in PCa. Thirteen USP13-targeted microRNAs (miRNAs) were identified using ENCORI software (Fig. 9A). The correlations between the expression of USP13 and candidate miRNAs in PCa were analyzed based on TCGA_PRAD, and the prognostic values of miRNAs for the overall survival of PCa patients were examined using KM analysis. As shown in Fig. 9B, hsa-miR-19a-3p (miR-19a for short), hsa-miR-19b-3p (miR-19b for short) and hsa-miR-485-5p (miR-485 for short) was found to be significantly associated with poor OS of PCa patients and negatively correlated to USP13. Additionally, the expression of 13 candidate miRNAs in normal and PCa tumor tissues was analyzed, and miRNAs upregulated in PCa tumors are shown in Fig. 9C. Taken together, miR-19a/19b and miR-485 were selected as USP13-targeted miRNAs which also possessed some prognostic values in PCa. The upstream lncRNAs were then predicted by ENCORI, and common interacted lncRNAs between 2 or 3 miRNAs were analyzed and shown in a Venn diagram (Fig. 9D). Collectively, the lncRNA–miRNA-mRNA triple network and correlations are shown in Fig. 9E.

**Fig. 10:**
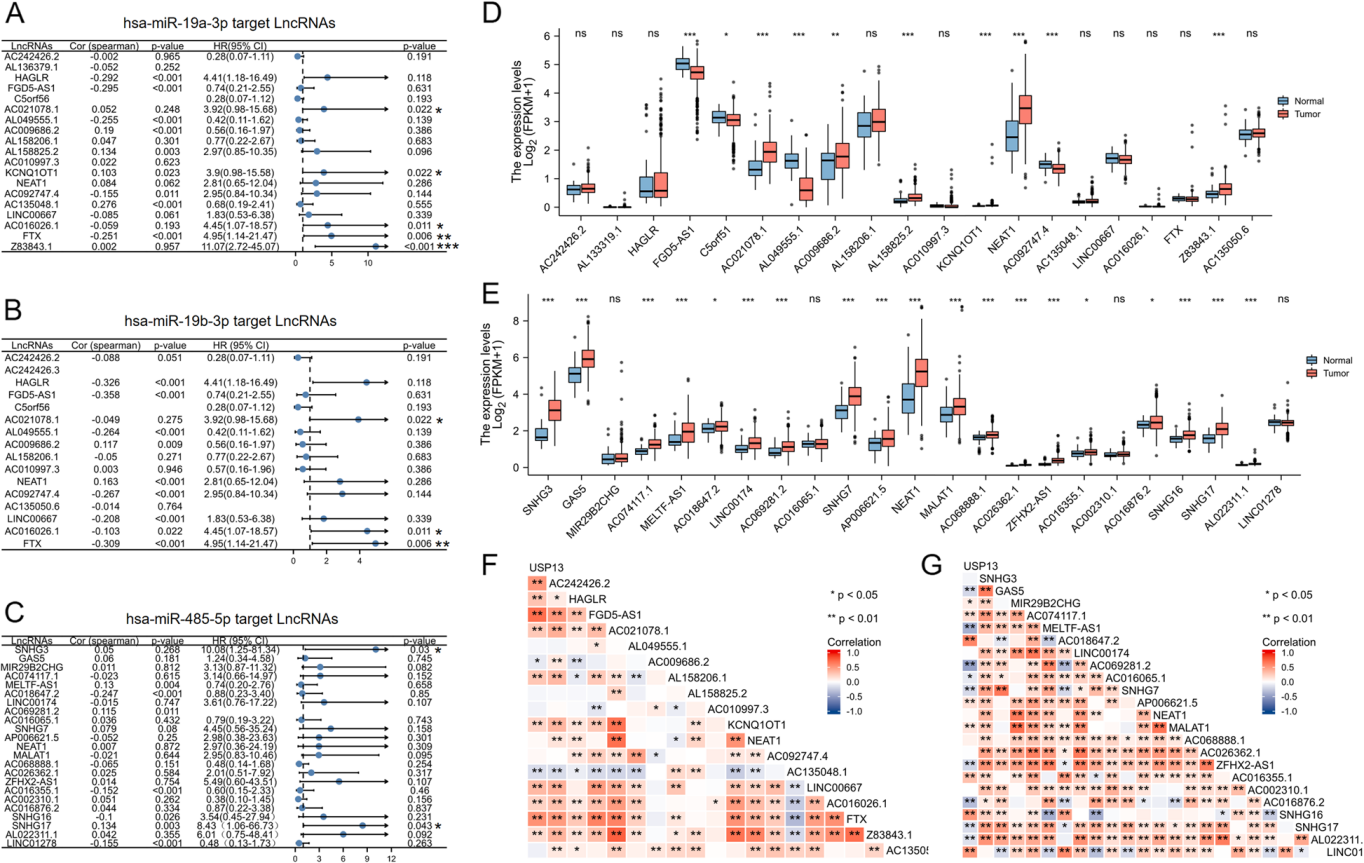
Expression analysis and survival analysis for upstream lncRNAs in PCa

The expression correlations between LncRNAs and miRNAs, and the predictive roles of LncRNAs in PCa were examined and listed in Fig. 10A to C. Among the candidate lncRNAs, Z83843.1 and KCNQ1OT1 targeted by miR-19a, AC021078.1, AC016026.1 and FTX targeted by both miR-19a and miR-19b, and SNHG3 and SNHG17 targeted by miR-485 were found to be related to the OS of PCa patients. Next, the expression levels of all candidate lncRNAs between normal and tumor tissues were analyzed and compared (Fig. 10D and E). Subsequently, the correlations between the expression of candidate LncRNAs and USP13 were evaluated, and LncRNAs that positively or negatively correlated to USP13 are documented in Fig. 10F and G. All LncRNAs were subjected to three categories: LncRNAs which positively correlated to USP13 and negatively correlated to miRNAs, LncRNAs which had predictive potentials for OS of PCa patients, and LncRNAs which were highly expressed in PCa tumors (Table 4). We found that the LncRNAs crossed in two categories were FTX and AC021078.1 targeted by miR-19a/19b, and SNHG3, SNHG16, SNHG17, AC018647.2 and AC016355.1 targeted by miR-485. No lncRNAs were found to cross in all the three categories (Table 5). In addition, NEAT1 was the only common lncRNA targeted by miR-19a/19b and miR-485, indicating that NEAT1 was very likely to regulate USP13 expression through the ceRNA network.

**Table 4:** Analysis of potential LncRNAs participated in the ceRNA network targeting USP13

Positive correlations between expression of LncRNAs, miRNAs and USP13					
miR-19a/b targets			miR-485 targets		
	miR-19a/b	USP13		miR-485	USP13
HAGLR	negative ***	positive **	MELTF-AS1	positive **	negative **
FGD5-AS1	negative ***	positive **	AC018647.2	negative ***	positive **
AL049555.1	negative ***	(-)	AC069281.2	positive *	negative **
AC009686.2	positive ***	negative *	AC016355.1	negative ***	positive **
AC092747.4	negative *	(-)	SNHG16	negative *	positive **
FTX	negative ***	positive **	SNHG17	positive **	negative **
			LINC01278	negative ***	positive **
Positive predictive LncRNAs for OS					
miR-19a/b targets			miR-485 targets		
AC021078.1			SNHG3		
KCNQ1OT1			SNHG17		
AC016026.1					
FTX					
Z83843.1					
Highly expressed LncRNAs in PCa tumors					
miR-19a/b targets			miR-485 targets		
AC021078.1			SNHG3	SNHG7	AC016355.1
AC009686.2			GAS5	AP006621.5	AC016876.2
AL158825.2			AC074117.1	NEAT1	SNHG16
KCNQ1OT1			MELTF-AS1	MALAT1	SNHG17
NEAT1			AC018647.2	AC068888.1	AL022311.1
Z83843.1			LINC00174	AC026362.1	
			AC069281.2	ZFHX2-AS1	

* indicates *p* < 0.05, ** indicates *p* < 0.01, *** indicates *p* < 0.001

**Table 5:** Potential LncRNAs of ceRNA network

LncRNAs which crossed two categories	
miR-19a/b targets	FTX, AC021078.1
miR-485 targets	SNHG3, SNHG16, SNHG17, AC018647.2, AC016355.1

A. USP13-miRNA network predicted by ENCORI. B. Prognostic values of candidate miRNAs in PCa patients analyzed using KM plotters. C. Expression of candidate miRNAs between normal and PCa tumor tissues based on TCGA. D. Venn diagram suggesting the potential interacting LncRNAs of miR-19a/b and miR-485. E. USP13-miRNA–lncRNA network analyzed by ENCORI. * indicates *p* < 0.05, ** indicates *p* < 0.01, *** indicates *p* < 0.001.

Analysis of the expression of miR-19a-targeted lncRNAs (A), miR-19b-targeted lncRNAs (B) and miR-485-targeted lncRNAs (C) and the overall survival of PCa patients by KM plotters. Expression of miR-19a/b-targeted LncRNAs (D) and miR-485-targeted LncRNAs (E) between normal and PCa tumor tissues based on TCGA. (F). Correlation analysis between the expression of miR-19a/b-targeted lncRNAs and USP13 in PCa. (G). Correlation analysis between the expression of miR-485-targeted lncRNAs and USP13 in PCa. * indicates *p* < 0.05, ** indicates *p* < 0.01, *** indicates *p* < 0.001.

## Discussion

Accumulating evidence has identified USP13 as a hallmark of cancer suppression, as it directly interacts with P53 and PTEN and sustains protein stability through its ubiquitinating attribute. However, studies have also shown that USP13 may act as an oncogene by promoting Mcl-1 stability in cervical cancer [36], and USP13 also drives the progression of ovarian cancer by promoting cancer metabolism [37]. In addition, USP13 is reported to participate in DNA damage response [25] and STING signaling [26] and could thereby be a potential target to facilitate the efficacy of DDR inhibitors and immunotherapy. To date, no further studies have been carried out to demonstrate the potential role of USP13 in anticancer therapy, nor revealing that targeting USP13 may synergize with DDR inhibitors or ICIs. In addition, its biological functions in prostate cancer still remain to be elucidated. In the current study, our data showed that USP13 is highly expressed in PCa tumors and that high expression of USP13 indicates poor survival of PCa patients. Moreover, our study supported a promising therapeutic role of USP13 in prostate cancer: small molecular inhibitors targeting USP13 may improve the antitumor effects of DDR inhibitors and enhance innate immunity and immune infiltration, which might provide a new chance for immunotherapy to PCa patients.

Through the present study, we concluded that USP13 potentially modulates the tumor microenvironment of prostate cancer through multiple mechanisms. As analyzed by Metascape, USP13 was identified to facilitate protein neddylation. Neddylation is a reversible covalent conjugation of the ubiquitin-like molecule neuronal precursor cell-expressed developmentally downregulated protein 8 (NEDD8) to a lysine residue of the substrate protein [38, 39], thereby modulating many crucial biological processes, including tumorigenesis [40, 41]. In addition, studies have confirmed that inhibition of the neddylation pathway influences a variety of important TME components, such as immune cells, cancer-associated fibroblasts (CAFs) and cancer-associated endothelial cells (CAEs) [42, 43]. Neddylation has been recognized as a therapeutic target for cancer therapy, as the highly selective small molecular inhibitor acting on NEDD8-activating enzyme (NAE), named MLN4924, has been developed and shown strong antitumor activity and well-tolerated toxicity in several preclinical studies [44–46]. Through anticipating neddylation process, USP13 potentially enhanced the activities of CAFs, CAEs and tumor-associated macrophages (TAMs), thereby promoting angiogenesis and tumor metastasis [42]. However, there is still much work to do to support this hypothesis.

USP13 was also reported to interact with STING, a primary mediator of innate immune signaling, and subsequently prevent the recruitment of TBK1 to the signaling complex, thereby negatively regulating innate immunity [26]. In this sense, USP13 inhibition may activate the STING pathway along with innate immunity, which may provide a synergetic effect with ICIs. However, controversial evidence also revealed that USP13 stabilizes STAT1 [47], indicating that USP13 is a mediator for the activation of interferon (IFN) signaling, while IFNs play important roles in antiviral and antitumor activities. Another important function of USP13 is that it promotes the DNA damage response in ovarian tumor cells by recruiting the BRCA1/RAP80 complex to DNA damage sites. We also observed a positive correlation between the expression of USP13 and DDR genes in prostate cancer samples. Deficiency in DNA damage repair may lead to the activation of cGAS-STING signaling and innate immunity [48]. Hence, targeting USP13 may lead to DDR deficiency and high MSI as well as activation of innate immune and antitumor immunity, and may promote a synergetic effect with DDR inhibitors and ICIs. Nonetheless, the role of USP13 in innate immune and antitumor immunity still needs to be further studied.

Our present study revealed that USP13 may participate in multiple signaling pathways which may lead to tumorigenesis, such as PI3K/AKT signaling, Wnt signaling, TGF-beta signaling and EGFR signaling. We also found that the expression of USP13 significantly correlated with AR coactivators, AR target genes and PCa-related genes. These findings indicate that USP13 may serve as a strong driver in promoting carcinogenesis of PCa. Activation of PI3K signaling is a common event in prostate cancer that modulates the initiation, progression and therapeutic resistance of PCa [49–51]. PI3Ks are a family of lipid kinase enzymes and are divided into three classes, namely, Class I (subdivided into Class IA and IB), Class II, and Class III. Among them, the class IA PI3K subunit PIK3CA is a primary molecule that activates the PI3K cascade. Mutations and amplifications of PI3KCA are highly prevalent in human cancers and occur in 4 and 9% of PCa cases [52]. Evidence suggests that PIK3CA is a genetic driver for PCa. We found that USP13 gene amplification is common in cancers, and USP13 significantly co-expressed with PIK3CA in PCa tissues. The Wnt family controls cell growth, organ development, tissue homeostasis and stem cell renewal in human organs and is important for prostate development [53]. Activation of Wnt signaling leads to the stabilization and accumulation of β-catenin, which subsequently transcriptionally upregulates Wnt target genes such as MYC and AXIN2 [54]. In addition, β-catenin can directly interact with AR in the nucleus to facilitate the transcriptional activity of AR-targeted genes [55, 56]. PI3K and Wnt signaling cascades can cooperate to induce the localization of β-catenin and interactions of β-catenin with other transcription factors, such as FOXO3a [57] and SOX4 [58]. The linkage of USP13 to these PCa-related signaling pathways uncovers the underlying mechanisms of USP13 in cancers and provides solid evidence to support its role in facilitating AR activity.

Having discovered the cancer-driven potential of USP13 in PCa, we then sought to determine whether there is an effective inhibitor of USP13 to suppress its oncogenic functions. Specific and potent autophagy inhibitor-1 is a potent small molecule inhibitor of autophagy that also selectively regulates the deubiquitinating activities of USP10 and USP13 [21]. Spautin-1 has been identified to control P53 activity by targeting USP10 and USP13, and the antitumor function of spautin-1 has been confirmed in human cancer models [21, 59, 60]. Liao et al. reported that spautin-1 inhibited EGFR signaling and can be considered a potential PCa therapy [61]. However, they noted that the anti-tumour function of spautin-1 in PCa is independent of USP10, USP13 and autophagy. In our study, USP13 was found to be possibly involved in EGFR signaling, indicating that USP13 inhibition may interrupt the signaling cascade; however, the effect of USP13 inhibition (genetically or pharmaceutically) on the malignancy phenotype of PCa cells and the expression of signaling components still need further validation.

Noncoding RNAs play critical roles in regulating cancer progression, and it has been well acknowledged that long noncoding RNAs can regulate the expression of mRNAs via competitive endogenous RNA mechanisms. In this study, we analyzed the ncRNA-mediated ceRNA network of USP13 in PCa. The USP13-targeted miRNAs were predicted, and the prognostic and expression profiles of all miRNA candidates in PCa were subsequently analyzed. Combined, miR-19a/b and miR-485 were selected as potential miRNAs. miR-19a and 19b belong to the miR-17-92a cluster, which is frequently described as oncogenes in multiple cancers [62]. The lncRNAs competitively interacting with miR-19a/b and miR-485 were then analyzed, and the expression correlations between lncRNA, miRNA and USP13, prognostic potential and expression profiles of lncRNAs in PCa was analyzed to identify the candidate lncRNAs. Our data showed that NEAT1 was the only common lncRNA that may interact with both miR-19a/b and miR-485. Oncogenetic properties of NEAT1 in PCa have been documented as modulating bone metastasis [63], cell proliferation [63] and cellular DNA damage repair [64]; however, none of the studies mentioned NEAT1-USP13 regulation. Our study indicated a potential mechanism through which USP13 gene expression was regulated in prostate cancer, although the ceRNA network needs to be further validated in cell line and animal models.

There are some limitations in this study. First, most of the conclusions were basing on data mining and bioinformatic analysis, therefore, more work needs to do to validate our hypothesis. Second, the size of our cohort included in this study was too small, a large cohort of patients with clinical tissue specimens was needed to make a better statistical analysis regarding the clinical significance of USP13 in prostate cancer. Third, although we tried to identify the prognostic value of USP13 gene in prostate cancer, the results were mostly basing on gene expression of USP13 in PCa samples. It is worth investigating the protein expression of USP13 with clinical outcomes of PCa patients. Last, to further examine the clinical significance of USP13, it is of great value to analyze the effects of small molecular inhibitors against USP13, such as spauntin-1, on tumor formation and growth in in vitro and in vivo PCa models.

To conclude, our study uncovered the prognostic value and carcinogenic role of USP13 in prostate cancer and showed that overexpression of USP13 may lead to activation of cancer-driving signaling pathways, such as PI3k, Wnt and AR. Additionally, USP13 inhibition potentially suppresses DNA damage repair and protein neddylation of PCa cells, thereby facilitating the cellular response to stimuli and the modification of TME. Novel therapies targeting USP13 may synergize with DDR inhibition and immunotherapy and provide an extra antitumor effect against PCa and CRPC. Nonetheless, much work including cell line, animal models and RNA sequencing still need to be done to validate the hypothesis in the future.

## Supplementary Information

**Additional file 1.**

## Data Availability

The datasets used and/or analyzed during the current study are available in the following repositories, GTEx database (https://gtexportal.org/), TCGA database (https://portal.gdc.cancer.gov/), GEO database (https://www.ncbi.nlm.nih.gov/geo/), accession numbers: GSE137829, GSE141445, GSE143791, GSE150692 and GSE172301.
