# Supplementary material for: ncRNA-mediated overexpression of ubiquitin-specific proteinase 13 contributes to the progression of prostate cancer via modulating AR signaling, DNA damage repair and immune infiltration
Source: BMC Cancer. 2022 Dec 23;22:1350. doi: 10.1186/s12885-022-10424-7 (PMC9784269; doi:10.1186/s12885-022-10424-7)
Supplement: Supplementary file 1 — Additional file 1. [file 12885_2022_10424_MOESM1_ESM.pdf]

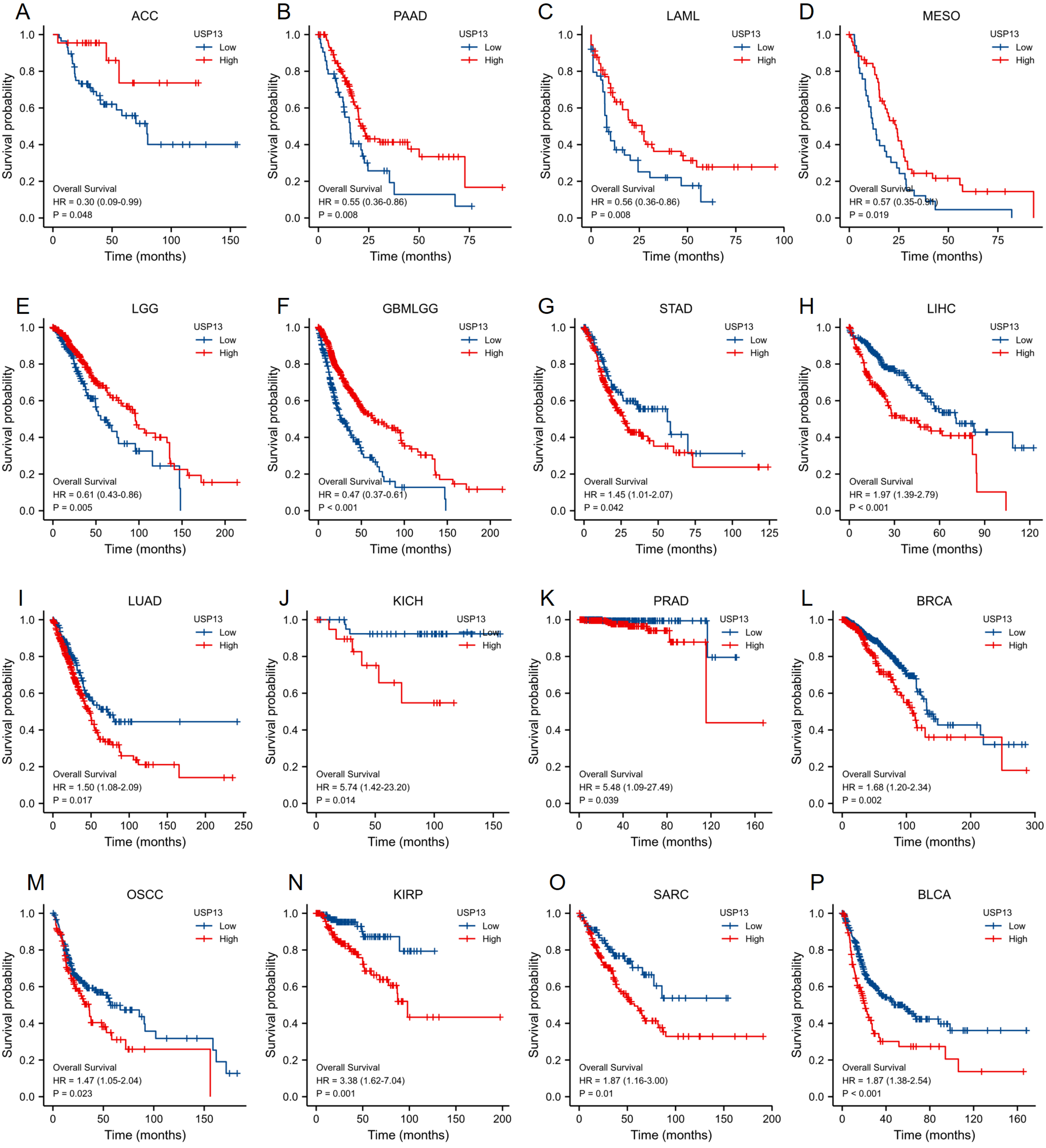

Supplementary Figure 1. Analysis of USP13 expression with overall survival of patients in multiple cancer types by KM plotters.

High expression of USP13 was correlated with optimistic OS of patients with ACC (Adrenocortical carcinoma) (A), PAAD (Pancreatic adenocarcinoma) (B) and LAML (Acute Myeloid Leukemia) (C), and with poor OS of patients with MESO (Mesothelioma) (D), LGG (Brain Lower Grade Glioma) (E), GBMLGG (Glioblastoma multiforme and Brain Lower Grade Glioma) (F), STAD (Stomach adenocarcinoma) (G), LIHC (Liver hepatocellular carcinoma) (H), LUAD (Lung adenocarcinoma) (I), KICH (Kidney Chromophobe) (J), PRAD (Prostate adenocarcinoma) (K), BRCA (Breast invasive carcinoma) (L), OSCC (Oral Squamous Cell Carcinoma) (M), KIRP (Kidney renal papillary cell carcinoma) (N), SARC (Sarcoma) (O) and BLCA (Bladder Urothelial Carcinoma) (P).

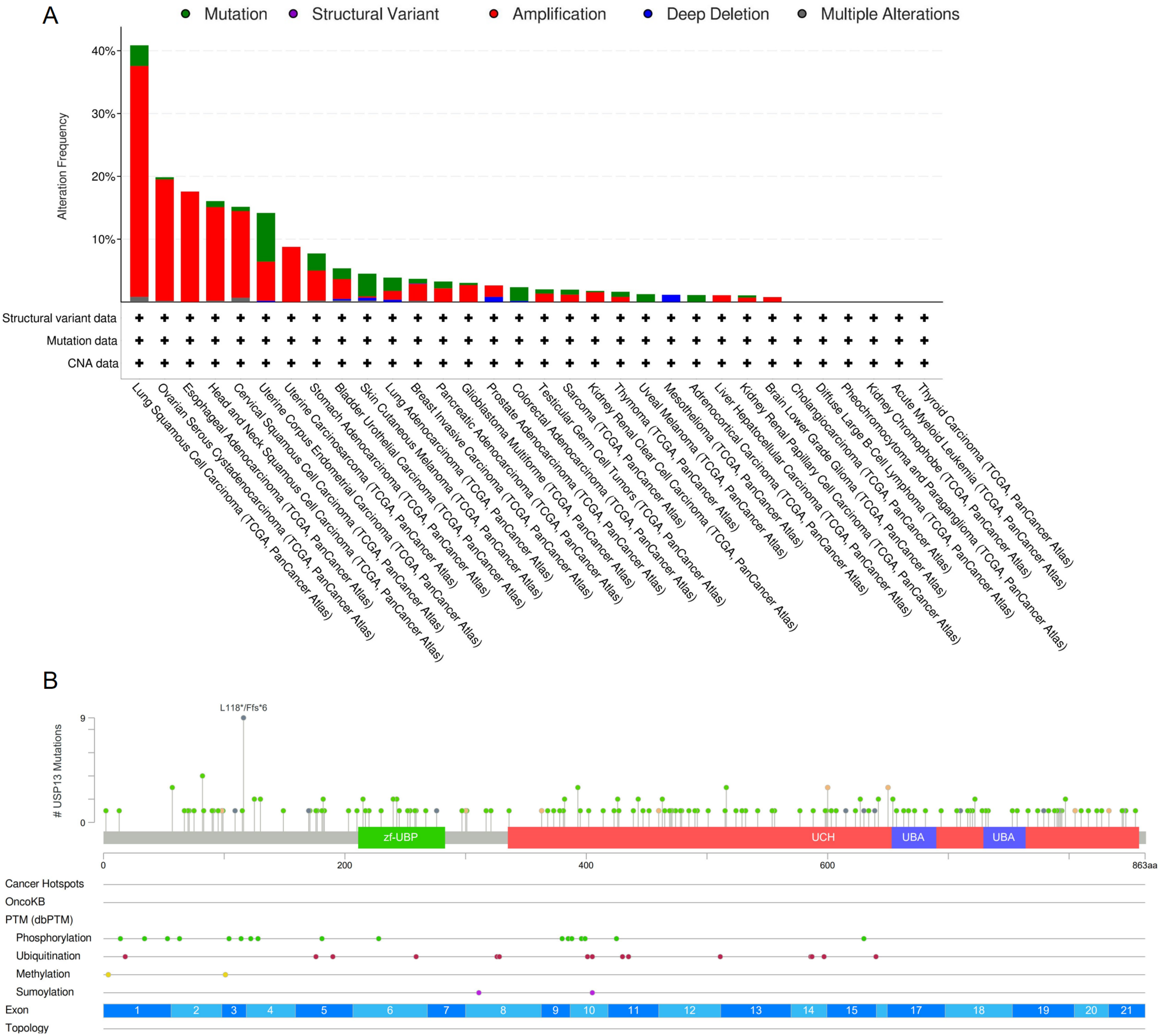

Supplementary Figure 2. Mutation, amplification and alterations of USP13 gene in cancers.

A. Amplification of USP13 gene is common in cancers. (B) The protein structure along with the phosphorylation, ubiquitination and methylation sites of USP13.

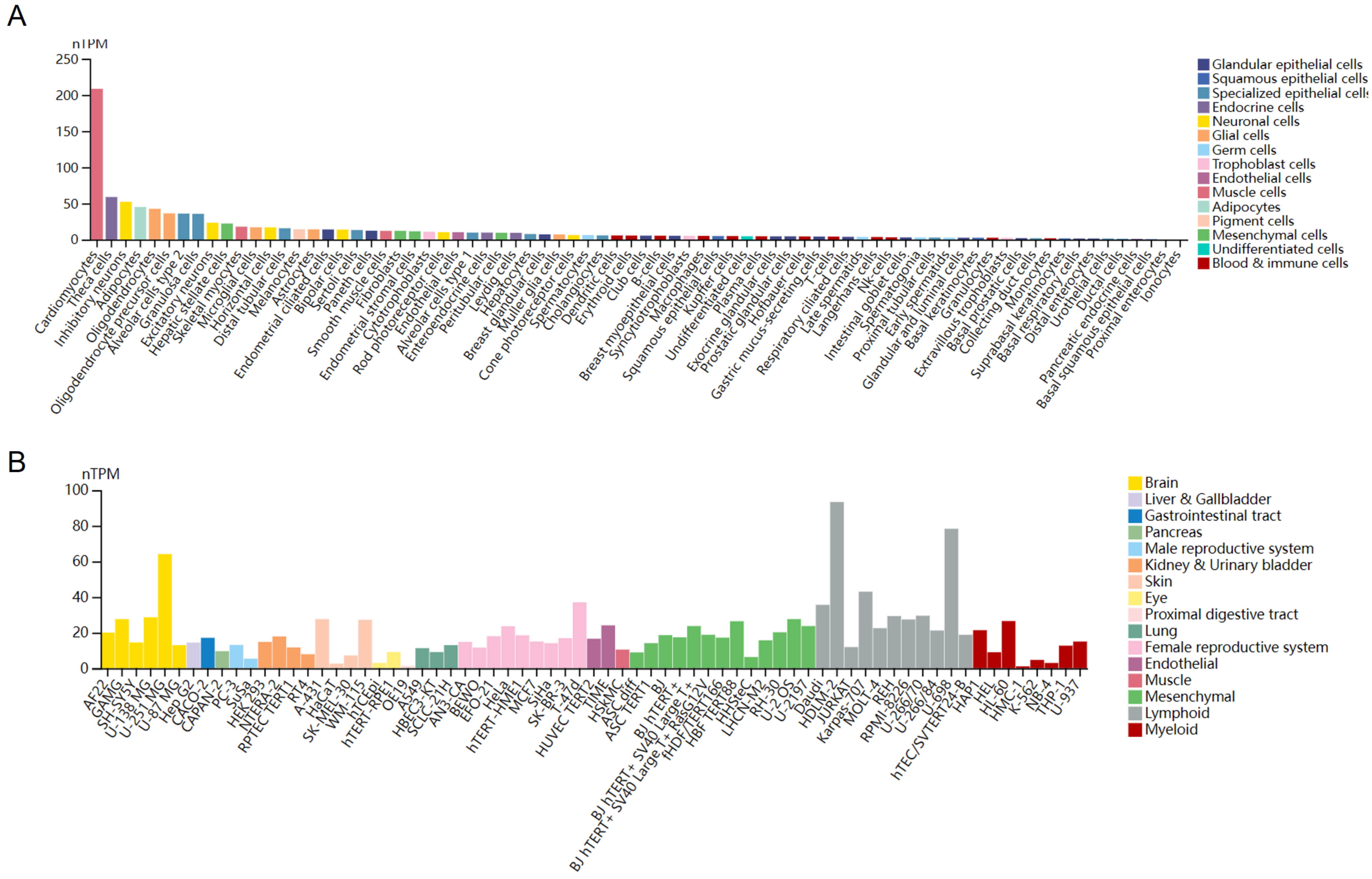

Supplementary Figure 3. Expression of USP13 in single cell types (A) and cell lines (B) by The Human Protein Atlas

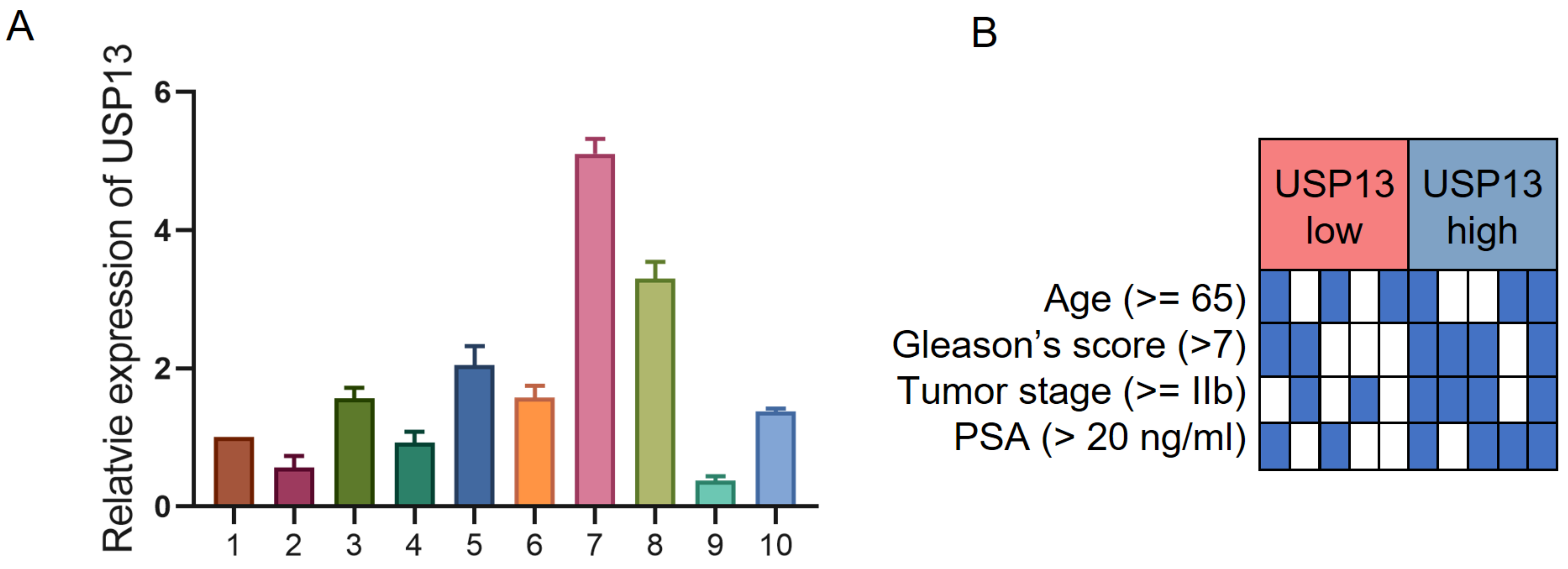

Supplementary Figure 4. The USP13 gene expression and clinicopathological characteristics of the patients.

A. USP13 gene expression was detected in clinical tumor tissue samples from 10 PCa patients by qRT-PCR. B, 10 PCa patients were divided into two group by the median level of USP13 expression, and clinicopathological characteristics (age, Gleason's score, T stage and PSA level) of each patient were recorded accordingly.

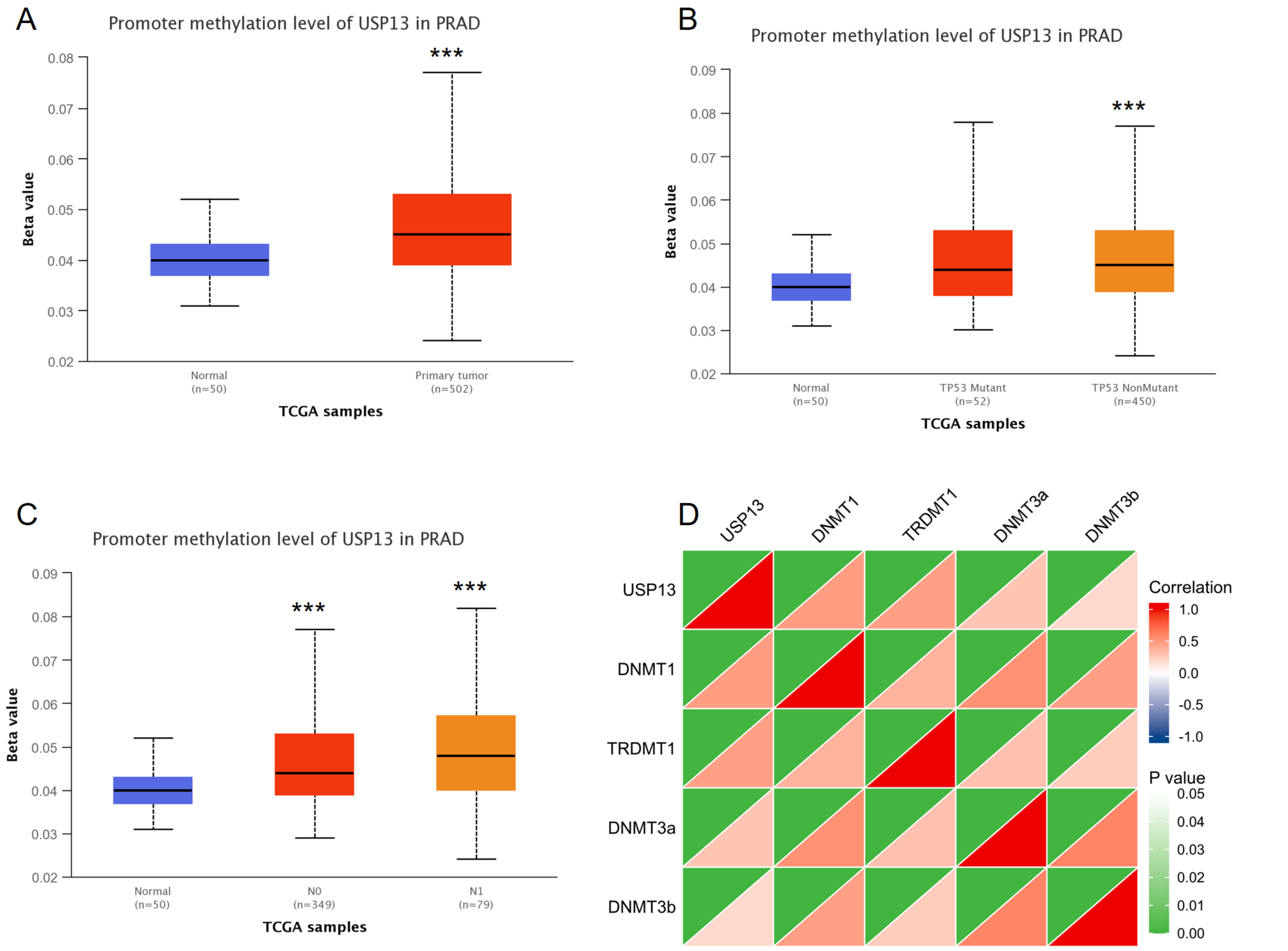

Supplementary Figure 5. Methylation status of USP13 in PCa.

A. Methylation status of USP13 promoter between normal and PCa tumor tissues. B. Methylation status of USP13 promoter between normal tissues, tumor tissues with or without TP53 mutation. C. Methylation status of USP13 promoter between normal tissues, tumor tissues with or without lymph node metastasis. D. Associations between expression of USP13 and methyltransferases. * indicates $p < 0.05$, ** indicates $p < 0.01$, *** indicates $p < 0.001$.

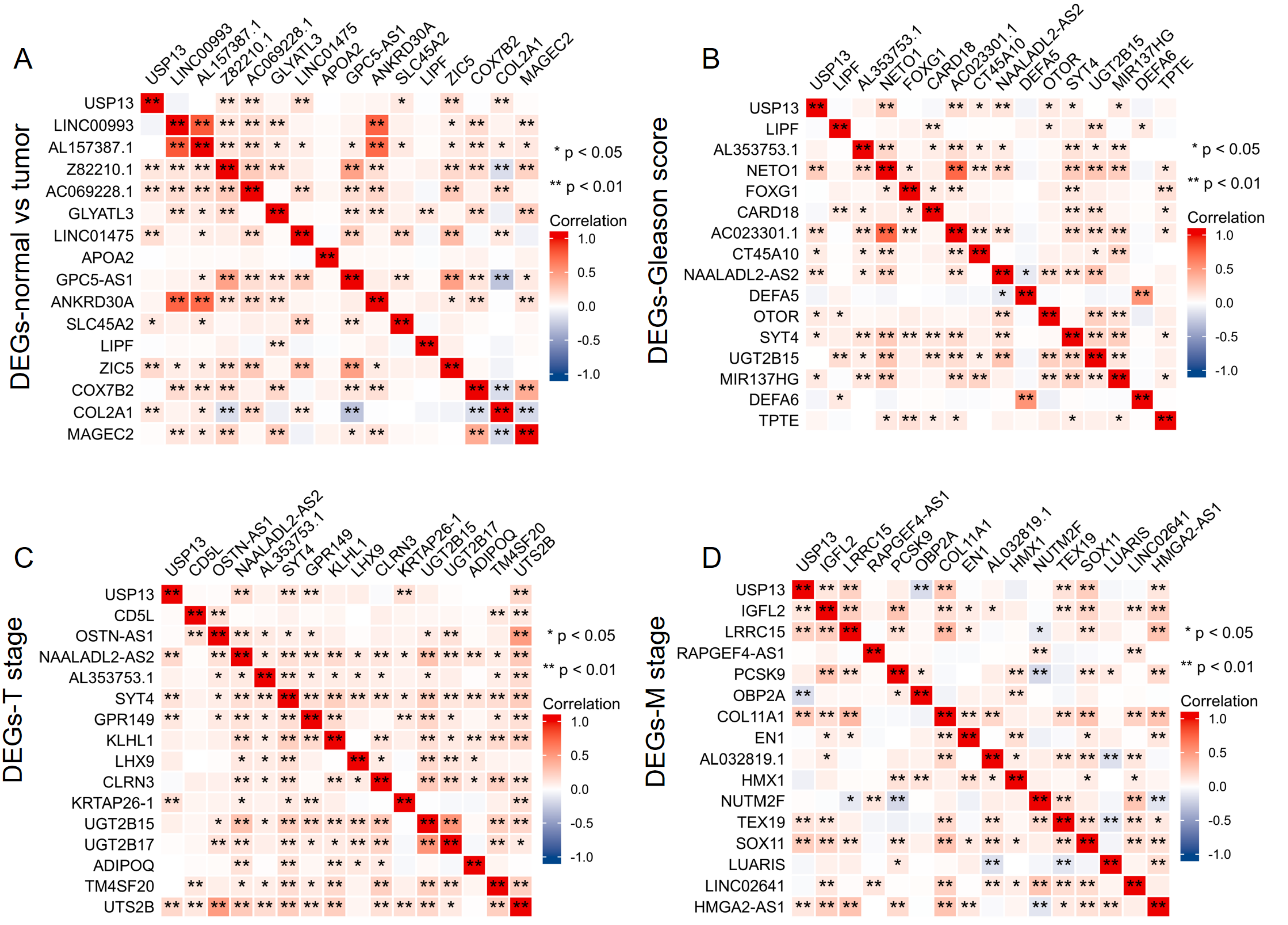

Supplementary Figure 6. Association between USP13 Expression and PCa progression.

Association between expression of USP13 and top 15 DEGs of normal samples against PCa tumor tissues (A), low Gleason's score (6 & 7) against high Gleason's score (8 & 9 & 10) (B), tumors with T2 stage against T4 stage (C) and tumors with distant metastasis against those without distant metastasis (D). * indicates $p < 0.05$, ** indicates $p < 0.01$.

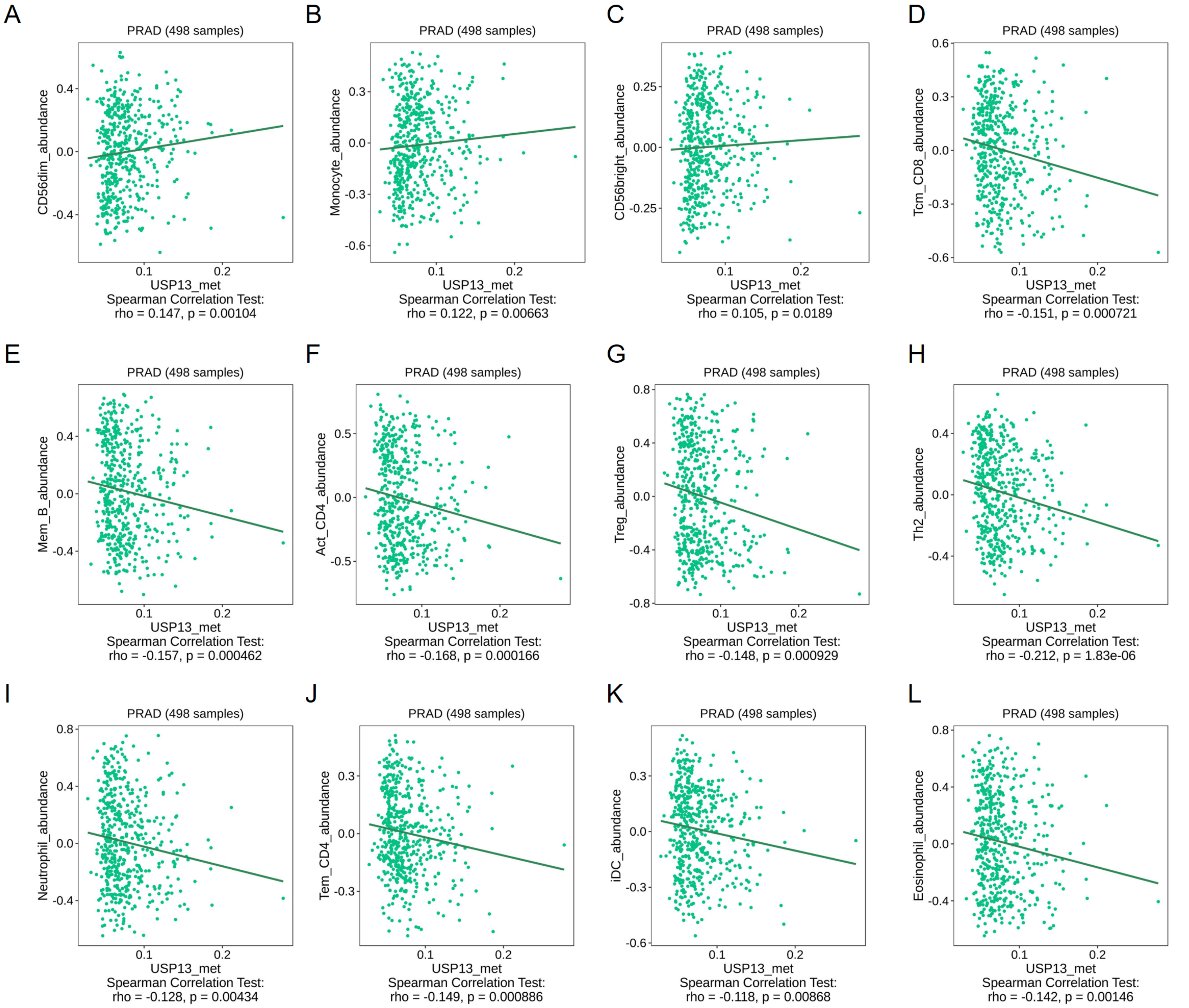

Supplementary Figure 7. Associations between the methylation status of USP13 with immune infiltrates in PCa by TISIDB.

Associations between methylation of USP13 and abundance of CD56dim natural killer cell (A), Monocyte (B), CD56bright natural killer cell (C), central memory CD8 T cell (Tcm_CD8) (D), memory B cell (Mem_B) (E), activated CD4 T cell (Act_CD4) (F), Regulatory T cell (Treg) (G), Type 2 T helper cell (Th2) (H), Neutrophil (I), effector memory CD4 T cell (Tem_CD4) (J), immature dendritic cell (iDC) (K) and Eosinophil (L).
